# Four New Chromones from the Endophytic Fungus *Phomopsis asparagi* DHS-48 Isolated from the Chinese Mangrove Plant *Rhizophora mangle*

**DOI:** 10.3390/md19060348

**Published:** 2021-06-19

**Authors:** Chengwen Wei, Chunxiao Sun, Zhao Feng, Xuexia Zhang, Jing Xu

**Affiliations:** 1 School of Chemical Engineering and Technology, Hainan University, Haikou 570228, China; weichengwen@hainanu.edu.cn (C.W.); fz329077397@163.com (Z.F.); zhangxuexia@hainanu.edu.cn (X.Z.); 2 School of Medicine and Pharmacy, Ocean University of China, Qingdao 266003, China; scx@stu.ouc.edu.cn

**Keywords:** mangrove endophytic fungi, *Phomopsis* sp., chromenones, immunosuppressive activity

## Abstract

Four new chromones, phomochromenones D–G (**1**–**4**), along with four known analogues, diaporchromone A (**5**), diaporchromanone C (**6**), diaporchromanone D (**7**), and phomochromenone C (**8**), were isolated from the culture of *Phomopsis asparagi* DHS-48 from Chinese mangrove *Rhizophora mangle*. Their structures were elucidated on the basis of comprehensive spectroscopic analysis. The absolute configurations of **1** and **4** were assigned on the basis of experimental and calculated electronic circular dichroism (ECD) data, and those of enantiomers **2** and **3** were determined by a modified Mosher’s method and basic hydrolysis. To the best of our knowledge, phomochromenones D–F (**1**–**4**) possessing a 3-substituted-chroman-4-one skeleton are rarely found in natural sources. Diaporchromone A (**5**) showed moderate to weak immunosuppressive activity against T and/or B lymphocyte cells with IC_50_ of 34 μM and 117 μM.

## 1. Introduction

**Figure 1:**
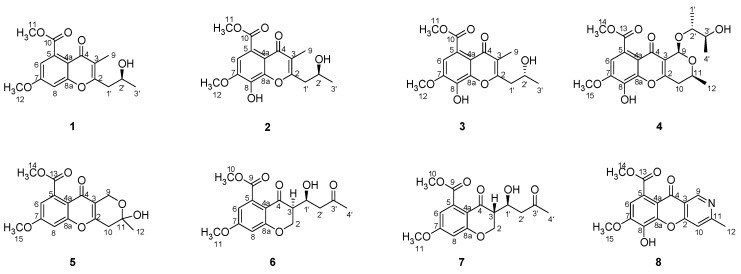
The chemical structures of compounds **1**–**8**.

Marine-derived fungi are morphologically and physiologically adapted to harsh environmental stresses, such as high salinity, high temperature, extreme tides, oxygen pressure, high humidity, and light and air limitations, which have increasingly attracted the attention of both pharmaceutical and natural product chemists in recent decades [1,2]. Fungi colonized in mangrove forests, which comprise the second largest ecological group of marine fungi, have especially adapted their metabolic mechanisms to the unique properties of the marine environment via the generation of a large variety of structurally unprecedented and biologically interesting metabolites of pharmaceutical importance [3,4,5]. One fungal genus which is especially productive with regard to the accumulation of a diverse array of mostly bioactive compounds is *Phomopsis*. Chemical investigation of this fungal genus has resulted in the discovery of over 70 potentially bioactive secondary metabolites, such as subintestinal vessel plexus (SIV) accelerator phomopsis-H76 A [6], cytotoxic phomopchalasins B and C [7], mycoepoxydiene [8], dicerandrols [9], antibiotic phomoxanthone A [10], phomodiol [11], phompsichalasin [12], antimicrotubule phomosidin [13], and anti-inflammatory phomol [14]. As part of our ongoing investigation on bioactive metabolites from mangrove endophytic fungi [15,16,17,18,19], *Phomopsis asparagi* DHS-48 was isolated from a fresh root of the mangrove plant *Rhizophora mangle*. Four new chromones (**1**–**4**), and five known compounds, including diaporchromone A (**5**) [20], diaporchromanone C (**6**) [20], diaporchromanone D (**7**) [20], and phomochromenone C (**8**) [21] (Figure 1) were isolated from the EtOAc extract of *P. asparagi* after fermentation on a solid rice medium containing sea salt. Herein, we report the isolation, structural elucidation, and exploration on the biological activities of compounds **1**–**8**.

## 2. Results and Discussion

**Figure 2:**
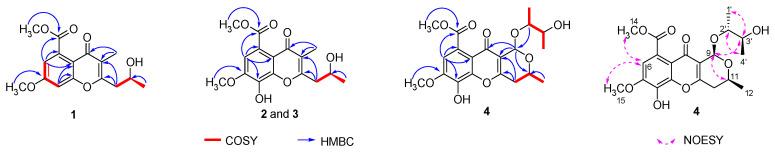
Selected 2D NMR of compounds **1**–**4.**

**Figure 3:**
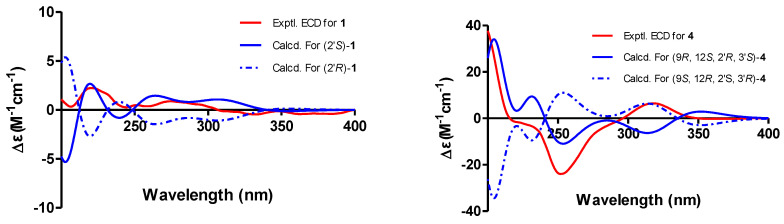
Experimental and calculated ECD spectra of **1** and **4.**

Phomochromenone D (**1**), a white amorphous powder, has the molecular formula C_16_H_19_O_6,_ established by HR-ESIMS (*m*/*z* 307.1139, calcd. for [M+H]^+^ 307.1182), implying eight degrees of unsaturation. The UV absorption maxima at 219, 245, 295 nm indicated that **1** could be a chromone derivative. The 1D NMR data of **1** (Table 1) indicated that six of the eight units of unsaturation came from four carbon–carbon double bonds and two carbonyls. Therefore, the other two units of unsaturation come from two rings. The ^1^H NMR spectrum of **1** showed the presence of two meta-coupled aromatic protons at *δ*_H_ 6.92 (d, *J* = 2.4 Hz, H-6) and *δ*_H_ 7.10 (d, *J* = 2.4 Hz, H-8), one methine at *δ*_H_ 4.25 (m, H-2′), one methylene at *δ*_H_ 2.92 (dd, *J* = 14.0, 8.0 Hz, H_a_-1′) and 2.60 (dd, *J* = 14.0, 5.1 Hz, H_b_-1′), and two methyl at *δ*_H_ 2.02 (s, H_3_-9) and *δ*_H_ 1.29 (d, *J* = 6.3 Hz, H_3_-3′), and two methoxy at *δ*_H_ 3.917 (s, H_3_-11) and *δ*_H_ 3.924 (s, H_3_-12),. The ^13^C NMR and DEPT spectra showed 16 carbon resonances corresponding to two sp^2^ methine (*δ*_C_ 114.52 and 102.3), ten sp^2^ quaternary (*δ*_C_ 165.1, 164.8, 159.5, 135.9, 119.1, 114.49, one carbonyl at *δ*_C_ 171.4, one conjugated carbonyl at *δ*_C_ 178.4), one oxygenated methine (*δ*_C_ 67.1), two methoxy (*δ*_C_ 57.0, 53.5), one methylene (*δ*_C_ 42.6), and two methyl (*δ*_C_ 23.7, 10.3) carbons. The HMBC correlation (Figure 2) from H-6 to C-5, C-7, and C-10, and from H-8 to C-7, C-4a and C-8a indicated the presence of the chromone moiety. Moreover, HMBC correlations from H_3_-9 to C-2, C-3 and C-4; and from H_2_-1′ to C-2 and C-3, suggested that a 2-hydroxypropyl group was attached to C-2 of the chromone core. The absolute configuration of C-2′ of **1** was determined by the comparison of its experimental and time-dependent density functional theory (TDDFT)-calculated electronic circular dichroism spectrum. The experimental ECD spectrum (CH_3_OH) for 2′*S*-**1** matched well with the calculated spectrum (Figure 3), which confirmed the unambiguous assignment of the absolute configuration of **1** as *S*, and the trivial name, phomochromenone D, was assigned.

**Figure 4:**
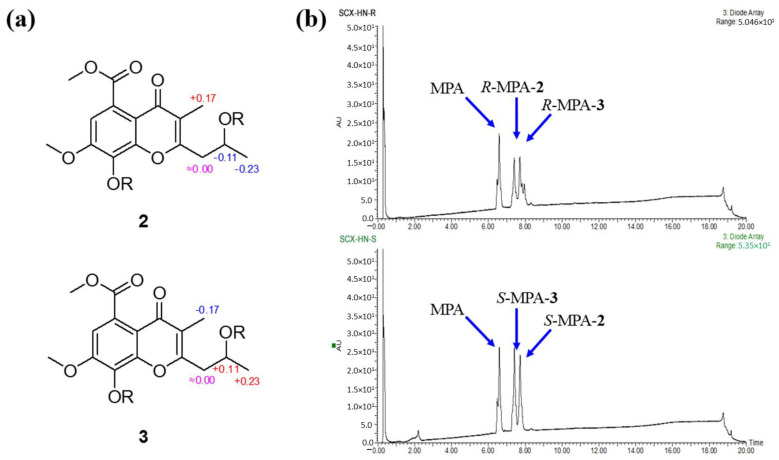
(**a**) Δ*δ* (=*δ_R_* − *δ_S_*) values for (*R*)- and (*S*)- MPA esters of **2** and **3**. (**b**) UPLC analysis profile of (*R*)- and (*S*)- MPA esters of **2** and **3** over a 20 min gradient as follows: T = 0.0, 5% B; T = 15.0, 95% B; T = 17.0, 100% B, T = 18.0, 100% B, and T = 18.1, 5% B, and T = 20.0, 5% B (A, MQ+0.2% HCOOH; B, MeOH+0.2% HCOOH).

Phomochromenones E (**2**) and F (**3**) were isolated as a mixture of two enantiomers and shared the same NMR data, ^1^H-^1^H COSY and HMBC spectra. Based on the HR-ESIMS ion detected at *m*/*z* 321.0989 (calcd. for [M−H]^−^, 321.0974), the mixture (**2**/**3**) had the same molecular formula of C_16_H_18_O_7_ (i.e., differing from that of **1** by an additional hydroxyl group). The ^1^H and ^13^C NMR spectrum clearly indicated that this hydroxyl group was attached at C-6. Supporting evidence for this assignment was obtained from the downfield chemical shifts of C-8 (*δ*_C_ 138.2, s) and the absence of the proton signal of H-6 in **2/3** (*δ*_H_ 7.10, d, *J* = 2.4 Hz for H-8 and *δ*_C_ 102.3, d for C-8 in **1**, respectively). Moreover, the presence of a hydroxyl group at C-8 was corroborated by the observed HMBC correlation from H-6 (*δ*_H_ 7.10, d, *J* = 2.4 Hz) to C-8, C-7 (*δ*_C_ 151.4), C-5 (*δ*_C_ 123.2), C-4a (*δ*_C_ 115.4), and C-10 (*δ*_C_ 172.4). However, the antipode rotation and ECD were detected, which suggested the mixture was not an optically pure compound. Since the chiral phase HPLC (CHIRALPAK IC) did not afford the separation of these two enantiomers, a modified Mosher’s experiment was performed to obtain its MPA esters. The products of Mosher’s reactions were subsequently analyzed by UPLC-ESI-MS through a RP-C_18_ chromatography column to afford two pairs of diastereomeric esters (*R*-MPA-**2**/*R*-MPA-**3** and *S*-MPA-**2**/*S*-MPA-**3**) (Figure 4). The spectral non-equivalences in ^1^H NMR chemical shifts between (*R*)- and (*S*)-MPA esters (Δ*δ* = *δ***_R_** − *δ***_S_**) indicated a 2′-*S* configuration for (+)-**2** (positive for H-9 and negative for H-3′) and a 2′-*R* configuration for (−)-**3** (negative for H-9 and positive for H-3′) (Figure 4). The (*R*)- and (*S*)-MPA esters of **2/3** was further separated by semi-prep. RP-C_18_ HPLC (40% CH_3_CN/H_2_O, 0.2% HCOOH) to afford *R*-MTPA-**2**/*S*-MTPA-**3** (*t*_R_ 18.8 min) and *R*-MPA-**3**/*S*-MPA-**2** (*t*_R_ 19.2 min). Compounds **2** and **3** were successfully obtained by chromatography after alkaline hydrolysis of *R*-MPA-**2**/*S*-MPA-**2** and *R*-MPA-**3**/*S*-MPA-**3**, respectively. Therefore, enantiomers **2** and **3** were successfully isolated and assigned the names phomochromenone E and phomochromenone F, respectively.

Phomochromenone G (**4**) was obtained as a white amorphous powder and had a molecular formula of C_20_H_24_O_9_, as determined by its HR-ESIMS (*m*/*z* 431.1314, calcd. for [M+Na]^+^ 431.1318), indicating nine degrees of unsaturation. The ^1^H NMR spectrum showed resonances for one singlet aromatic proton at *δ*_H_ 7.09 (s, H-6); four methine protons at *δ*_H_ 5.73(s, H-9), *δ*_H_ 4.40 (m, H-11), *δ*_H_ 3.60 (m, H-1′), and *δ*_H_ 3.54 (m, H-2′); one methylene at *δ*_H_ 2.77 (dd, *J* = 17.9, 3.7 Hz, H_a_-10) and 2.68 (dd, *J* = 17.9, 10.7 Hz, H_b_-10); three methyl at *δ*_H_ 1.37 (d, *J* = 6.2 Hz, H_3_-12), *δ*_H_ 1.13 (d, *J* = 6.0 Hz, H_3_-3′), and *δ*_H_ 1.19 (d, *J* = 6.0 Hz, H_3_-4′); two methoxy groups at *δ*_H_ 3.96(s, H_3_-15) and *δ*_H_ 3.87(s, H_3_-14). The ^13^C NMR and DEPT spectra showed 20 carbon signals, including a keto group, an ester carbonyl group, eight olefinic carbon signals (including four oxygenated carbons), three oxy-methines, one methylene, two methoxy group, and three methyl group. Comparison of the NMR data of **4** with those of phomochromenone B [21], previously isolated from endophytic fungus *Phomopsis* sp. HNY29-2B derived from mangrove plant *Acanthus ilicifolius* Linn, revealed that both compounds differed with regard to the nature of the side chain at C-1, where the hydroxyl group of the latter was replaced by the 3-hydroxybutan-2-yloxyl group of **3**. This was confirmed through ^1^H-^1^H COSY correlations of H_3_-4′/ H-1′/ H-2′/ H_3_-3′ and HMBC correlations (Figure 2) from H-9 to C-11(*δ*_C_ 63.9), C-4(*δ*_C_ 176.8), C-2(*δ*_C_ 166.7), and C-1′(*δ*_C_ 67.1). The relative configuration of **4** was based on the NOESY correlations as indicated in Figure 2. The NOESY correlations of H-9 to H-11, H_3_-1′ and H-3′; H-11 to H_3_-1′; and H_3_-4′ to H-2′ indicated that H-9, H-11, H_3_-1′, and H_3_-4′ were on the opposite side of the H_3_-12, H-2′, and H_3_-4′. The absolute configuration of **4** were also determined by comparing experimental and calculated electronic circular dichroism (ECD) spectra for the truncated model (9*R*, 12*S*, 2′*R*, 3′*S*)-**4** and the truncated model (9*S*, 12*R*, 2′*S*, 3′*R*)-**4** using time-dependent density functional theory (TDDFT). The theoretical spectrum of **4** showed an excellent fit with the experimental plot recorded in MeOH (Figure 3), which supported the absolute configuration to be 9*R*, 12*S*, 2′*R*, 3′*S*. Thus, the structure of **4** was determined and named phomochromenone F.

**Figure 5:**
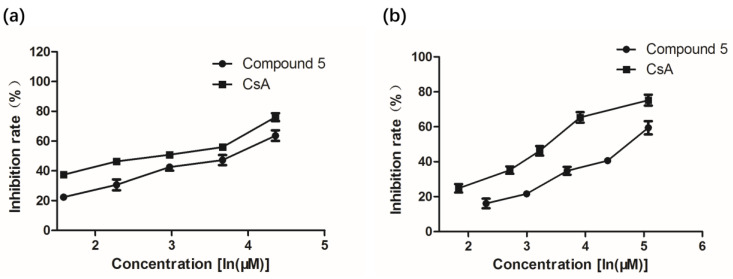
Effect of **5** on mouse splenocytes viability and proliferation. (**a**) Effect of **5** on the viability of T lymphocyte cells. (**b**) Effect of **5** on the viability of B lymphocyte cells. All values are expressed as mean ± SD. *n* = 3.

Our primary application of immunosuppressive activity screening indicated that a crude extract of *P. asparagi* DHS-48 showed strong inhibitory of splenic lymphocyte growth with IC_50_ of 6 μg/mL. An immunosuppressive assay showed that compound **5** exhibited moderate to weak inhibitory activity against ConA-induced T and LPS-induced B murine splenic lymphocytes *in vitro* with IC_50_ values of 34 and 117 μM, respectively (Table 2, Figure 5), whereas the other investigated compounds showed no obvious inhibitory effect. The cytotoxicity of **5** was tested in splenocyte cultures for 72 h using the tetrazolium salt-based CCK-8 assay. The results showed that it inhibited splenic lymphocyte growth with relatively lower toxicity (IC_50_ 47μM), at which the survival of normal splenic cells was slightly influenced in comparison with that of CsA (IC_50_ 11 μM). The results showed that compound **5** with a 1,3,4,10-tetrahydropyrano[4,3-b]chromene nucleus displayed significant immunosuppressive activity compared to compounds (**1**–**3**, **6**, **7**) with a chromone nucleus and compound **8** with a 10*H*-chromeno[3,2-c]pyridine nucleus. The additional 11-OH group in **5** is essential for its stimulated splenic lymphocyte inhibitory compared to compound **4**.

## 3. Materials and Methods

### 3.1. General Procedures

Specific rotations were obtained on a WYA-2S digital Abbe refractometer (Shanghai Physico-optical Instrument Factory, Shanghai, China). UV spectra were determined using a Shimadzu UV-2401 PC spectrophotometer (Shimadzu Corporation, Tokyo, Japan), while CD spectra were measured on a JASCO J-715 spectra polarimeter (Japan Spectroscopic, Tokyo, Japan). ^1^H, ^13^C and 2D NMR spectra were recorded on a Bruker AV 400 NMR spectrometer using TMS as an internal standard. High-resolution ESI-MS were performed on an LTQ Orbitrap XL instrument (Thermo Fisher Scientific, Bremen, Germany) using peak matching. TLC and column chromatography (CC) were carried out over silica gel (200–400 mesh, Qingdao Marine Chemical Inc., Qingdao, China), or a Sephadex-LH-20 (18−110 µm, Merck, Darmstadt, Germany), respectively. UPLC analysis (Waters Corporation, Milford, MA, USA) was recorded using a Waters system equipped in ESI mode on an Acquity UPLC H-Class connected to an SQ Detector 2 mass spectrometer using a BEH RP C_18_ column (2.1 × 50 mm, 1.7 µm, 0.5 mL/min). Semi-preparative HPLC was performed using a Waters equipped with a 2998 PDA detector (Waters Corporation, Milford, MA, USA) and a RP C_18_ column (YMC-Pack ODS-A, 10 × 250 mm, 5 μm, 3 mL/min).

### 3.2. Fungal Material

Endophytic fungus *Phomopsis asparagi* was isolated with PDA medium from the fresh root of the mangrove plant *Rhizophora mangle*, collected in October 2015 in Dong Zhai Gang-Mangrove Garden on Hainan Island, China. The fungus (strain no.DHS-8) was identified using a molecular biological protocol by DNA amplification and sequencing of the ITS region (GenBank Accession no.MT126606) [22]. A voucher strain was deposited at one of the authors’ laboratories (J.X.).

### 3.3. Extraction Isolation

The fungus was fermented onto auto autoclaved rice solid-substrate medium (thirty 1000 mL Erlenmeyer flasks, each containing 100 g of rice and 100 mL of 0.3% of saline water) and incubated for 28 days at 28 °C. In total, 140 flasks of culture were extracted three times with EtOAc and the filtrate was evaporated under reduced pressure to yield crude extract (65 g). The crude extract was partitioned with petroleum ether (PE), dichloromethane, ethyl acetate (EA), and n-butyl alcohol (BA). The dichloromethane fraction and ethyl acetate fraction were combined (30 g), then chromatographed on silica gel column chromatography using gradient elution with a CH_2_Cl_2_-MeOH mixture of increasing polarity (100:0–0:100, *v*/*v*) to afford 8 fractions (Fr. 1–Fr. 8). Fr. 2 was subjected to open silica gel CC using gradient elution with CH_2_Cl_2_-EtOAc (4:1–1:1, *v*/*v*) to yield fractions Fr. 2.1–2.6. Fr. 2.4 and Fr. 2.5 were purified by semi-preparative reversed-phase HPLC using MeOH-H_2_O (60:40, *v*/*v*) to afford **8** (5.0 mg) and **5** (4.8 mg), respectively. Fr. 3.4 was separated by silica gel CC using CH_2_Cl_2_-EtOAc (2:1, *v*/*v*) and was subsequently subjected to Sephadex LH-20 CC using MeOH as an eluent to give Fr. 3.4.5, followed by gradient elution MeOH-H_2_O (70:30–0:100, *v*/*v*) with semi-preparative reversed-phase HPLC to obtain **1** (1.2 mg) and a mixture of diaporchromanone C (**6**) and D (**7**) (5.1 mg). Purification of Fr. 4 was isolated using CC over silica gel CC using CH_2_Cl_2_-EtOAc (1:1, *v*/*v*) to afford Fr. 4.1–Fr. 4.6. Fr. 4.5 was purified using RP-18 with a MeOH−H_2_O (70:30, *v*/*v*) and then separated by semi-preparative reversed-phase HPLC with MeOH-H_2_O (50:50, *v*/*v*) to yield **2/3** (5.1 mg). Fr. 6.2, collected from Fr. 6, was subjected to silica gel CC with gradient elution of CH_2_Cl_2_-EtOAc (100:6–100:8, *v*/*v*) to give Fr. 6.2.1–Fr. 6.2.3. Fr. 6.2.2 was further separated by semi-preparative HPLC (MeOH-H_2_O, 55:45, *v*/*v*) to obtain **4** (3.0 mg).

### 3.4. Compound Characterization Data

Phomochromenone D (**1**): white amorphous powder (MeOH); [α]^20^_D_ − 13 (c 0.001, MeOH); UV (MeOH) λ_max_ 219, 245, 295 nm; ^1^H and ^13^C NMR data, see Table 1 and Table 2, respectively; HR-ESI-MS *m*/*z* 307.1179 [M+H]^+^ (calcd. for C_16_H_19_O_6_, 307.1182).

Phomochromenone E (**2**): yellow gum (MeOH); [α]^20^_D_ +17 (c 0.001, MeOH); UV (MeOH) λ_max_ 220, 245, 294 nm; ^1^H NMR data, see Table 1; HR-ESI-MS *m*/*z* 321.0989 [M−H]^−^ (calcd. for C_16_H_17_O_7_, 321.0974).

Phomochromenone F **(3)**: yellow gum (MeOH); [α]^20^_D_ − 19 (c 0.0008, MeOH); UV (MeOH) λ_max_ 220, 245, 294 nm; ^1^H NMR data, see Table 1; HR-ESI-MS *m*/*z* 321.0989 [M−H]^−^ (calcd. for C_16_H_17_O_7_, 321.0974).

Preparation of MPA Esters of **2/3** by Mosher’s Method. The mixture of compounds **2** and **3** (1.5 mg) was treated with (*R*)- or (*S*)-MPA (1.0 mg) with DCC (1.0 mg) and DMAP (0.3 mg) in anhydrous CH_2_Cl_2_ (0.5 mL). After being stirred at room temperature for 4 h at 0 °C, the solvent from the reaction mixture was removed in vacuo to furnish a residue, which was then subjected to semi-preparative RP-HPLC eluting with CH_3_CN-H_2_O (40:60, 0.2% HCOOH) to obtain *R*-MTPA-**2**/*S*-MTPA-**3** (*t*_R_ 18.8 min) and *R*-MPA-**3**/*S*-MPA-**2** (*t*_R_ 19.2 min).

(*R*)-MPA ester of **2**: yellow gum (MeOH); ^1^H NMR(400 MHz, CD_3_OD) *δ*_H_ 7.63–7.41 (m, 5H), 7.25–7.18 (m, 5H), 7.22 (s, 1H), 5.26 (s, 1H), 4.98 (m, 1H), 4.68 (s, 1H), 3.94 (s, 3H), 3.92 (s, 3H), 3.55 (s, 3H), 3.20 (s, 3H), 2.77 (m, 1H), 2.62 (m, 1H), 1.92 (s, 3H), 1.09 (d, *J* = 6.2, 3H); HR-ESI-MS *m*/*z* 619.2164 [M + H]^+^ (calcd. for C_34_H_35_O_11_, 619.2179). 

(*S*)-MPA ester of **2**: yellow gum (MeOH); 7.72–7.40 (m, 5H), 7.16 (s, 1H), 7.05–6.82 (m, 5H), 5.30 (s, 1H), 4.64 (s, 1H), 4.28 (m, 1H), 3.96 (s, 3H), 3.85 (s, 3H), 3.55 (s, 3H), 3.27 (s, 3H), 2.88 (m, 1H), 2.61 (m, 1H), 1.75 (s, 3H), 1.32 (d, *J* = 6.1, 3H); HR-ESI-MS *m*/*z* 619.2172 [M+H]^+^ (calcd. for C_34_H_35_O_11_, 619.2179).

(*R*)-MPA ester of **3:** yellow gum (MeOH); ^1^H NMR(400 MHz, CD_3_OD) *δ*_H_ 7.65–7.42 (m, 5H), 7.16 (s, 1H), 7.05–6.82 (m, 5H), 5.30 (s, 1H), 4.64 (s, 1H), 4.28 (m, 1H), 3.96 (s, 3H), 3.85 (s, 3H), 3.55 (s, 3H), 3.27 (s, 3H), 2.88 (m, 1H), 2.62 (m, 1H), 1.75 (s, 3H), 1.32 (d, *J* = 6.2, 3H); HR-ESI-MS *m*/*z* 619.2184 [M+H]^+^ (calcd. for C_34_H_35_O_11_, 619.2179). 

(*S*)-MPA ester of **3:** yellow gum (MeOH); ^1^H NMR(400 MHz, CD_3_OD) *δ*_H_ 7.63–7.41 (m, 5H), 7.25–7.19 (m, 5H), 7.22 (s, 1H), 5.26 (s, 1H), 4.98 (m, 1H), 4.68 (s, 1H), 3.94 (s, 3H), 3.92 (s, 3H), 3.55 (s, 3H), 3.20 (s, 3H), 2.77 (m, 1H), 2.60 (m, 1H), 1.92 (s, 3H), 1.09 (d, *J* = 6.2, 3H); HR-ESI-MS *m*/*z* 619.2157 [M+H]^+^ (calcd. for C_34_H_35_O_11_, 619.2179).

Preparation of phomochromenones E(**2**) and F**(3).** (*R*)- and (*S*)-MPA ester of **2** were combined (1.2 mg) and dissolved in 10% NaOH (1.0 mL) and stirred for 1 h at room temperature (rt). The reaction mixture was extracted with EtOAc repeatedly and the organic layer was evaporated in vacuo to give optically pure (+)-**2** (0.7 mg). Similarly, (−)-**3** (0.8 mg) was prepared from the alkaline hydrolysis of (*R*)- and (*S*)-MPA ester of **3** (totally 1.0 mg) in the same manner.

Phomochromenone G (**4**): white amorphous powder (MeOH); [α]^20^_D_ − 15 (c 0.001, MeOH); UV (MeOH) λ_max_ 205, 241, 303 nm; ^1^H and ^13^C NMR data, see Table 1 and Table 2, respectively; HR-ESI-MS *m*/*z* 431.1314 [M+Na]^+^ (calcd. for C_20_H_24_O_9_Na, 431.1318).

Diaporchromone A (**5**): yellow gum (CHCl_3_); [α]^20^_D_ − 40 (c 0.001, MeOH); UV (MeOH) λ_max_ 219, 294 nm; ^1^H NMR(400 MHz, CDCl_3_) *δ*_H_ 6.89(1H, d, *J* = 2.3Hz, H-6), 6.87(1H, d, *J* = 2.3 Hz, H-8), 4.72(2H, m, H-9), 3.98(3H, s, H-14), 3.90(3H, s, H-15), 2.86(1H, dd, *J* = 17.4 Hz, *J* = 2.8 Hz, 10-Ha), 2.76(1H, dd, *J* = 17.4 Hz, *J* = 9.8 Hz, 10-Hb), 1.63(3H, s, H-12); ^13^C-NMR (100 MHz, CDCl_3_) *δ*_C_ 173.7 (C-4), 169.8 (C-13), 163.2 (C-7), 158.4 (C-2), 158.0 (C-8a), 134.7 (C-5), 115.6 (C-3), 114.4 (C-4a), 113.0 (C-6), 101.7 (C-8), 95.4 (C-11), 57.5 (C-9), 56.2 (C-15), 53.2 (C-14), 37.1 (C-10), 29.4 (C-12). ESI-MS *m*/*z* 321.09 [M+H]^+^.

Diaporchromanone C (**6**): yellow gum (CHCl_3_); UV (MeOH) λ_max_ 215, 239, 275, 315 nm; ^1^H NMR(400MHz, CDCl_3_) *δ*_H_ 6.54(1H, d, *J* = 2.4 Hz, H = 6), 6.46(1H, d, *J* = 2.4 Hz, H = 8), 4.71(1H, dd, *J* = 11.6 Hz, *J* = 6.3 Hz, 2-Ha), 4.57(1H, dd, *J* = 11.6 Hz, *J* = 3.9 Hz, 2-Hb), 4.38 (1H, ddd, *J* = 8.5 Hz, *J* = 8.5 Hz, *J* = 2.9 Hz, H-1′), 3.94(3H, s, H-10), 3.84(3H, s, H-11), 2.66(1H, ddd, *J* = 8.5 Hz, *J* = 6.3 Hz, *J* = 3.9 Hz, H-3), 2.88(1H, dd, *J* = 18.1 Hz, *J* = 2.9 Hz, 2′-Ha), 2.71(1H, dd, *J* = 18.1 Hz, *J* = 8.5 Hz, 2′-Hb), 2.18(3H, s, H-4′); ^13^C NMR (125MHz, CDCl_3_) *δ*_C_ 210.0 (C-3′), 190.2 (C-4), 169.7 (C-9), 165.5 (C-8a), 164.0 (C-7), 136.3 (C-5), 111.7 (C-4a), 109.8 (C-6), 102.0 (C-8), 68.2 (C-2), 64.0 (C-1′), 56.1 (C-11), 53.1 (C-10), 50.6 (C-3), 47.7 (C-2′), 30.9 (C-4′). ESI-MS *m*/*z* 323.09 [M+H]^+^.

Diaporchromanone D (**7**): yellow gum (CHCl_3_); UV (MeOH) λ_max_ 215, 239, 275, 315 nm; ^1^H NMR(400MHz, CDCl_3_) *δ*_H_ 6.56(1H, d, *J* = 2.4 Hz, H = 6), 6.46(1H, d, *J* = 2.4 Hz, H = 8), 4.60(1H, dd, *J* = 11.5 Hz, *J* = 5.1 Hz, 2-Ha), 4.51(1H, ddd, *J* = 8.7 Hz, *J* = 5.1 Hz, *J* = 3.6 Hz, H-1′), 4.47 (1H, dd, *J* = 11.5 Hz, *J* = 9.6 Hz, 2-Hb), 3.93(3H, s, H-10), 3.85(3H, s, H-11), 2.96(1H, ddd, *J* = 9.6 Hz, *J* = 5.1 Hz, *J* = 5.1 Hz, H-3), 2.80(1H, dd, *J* = 17.3 Hz, *J* = 8.7 Hz, 2′-Ha), 2.74(1H, dd, *J* = 17.3 Hz, *J* = 3.6 Hz, 2′-Hb), 2.18(3H, s, H-4′); ^13^C NMR (125MHz, CDCl_3_) *δ*_C_ 208.8 (C-3′), 190.9 (C-4), 169.7 (C-9), 165.6 (C-8a), 164.1 (C-7), 136.3 (C-5), 112.3 (C-4a), 109.8 (C-6), 102.2 (C-8), 68.6 (C-2), 66.4 (C-1′), 56.1 (C-11), 53.1 (C-10), 49.6 (C-3), 46.8 (C-2′), 31.0 (C-4′). ESI-MS *m*/*z* 323.09 [M+H]^+^.

Phomochromenone C (**8**): yellow gum (MeOH); UV (MeOH) λ_max_ 206, 236, 308 nm; ^1^H NMR(400 MHz, CD_3_OD) *δ*_H_ 9.34(1H, s, H-1), 7.30(1H, s, H-4), 6.98(1H, s, H-8), 4.06(3H, s, H-14), 4.02(3H, s, H-13), 2.70(3H, s, H-3); ^13^C-NMR (125MHz, CD3OD) *δ*_C_ 176.3 (C-10), 171.7(C-12), 165.4 (C-3), 162.9 (C-4a), 153.9 (C-7), 150.1 (C-1), 146.6 (C-5a), 139.1 (C-6), 124.6 (C-9), 116.7 (C-10a), 115.4 (C-9a), 112.7 (C-4), 109.6 (C-8), 57.3 (C-14), 53.4 (C-13), 24.4 (C-11). ESI-MS *m*/*z* 316.12 [M+H]^+^.

### 3.5. Computational Analyses

Conformational analysis of the enantiomers of compounds **1** and **4** established by NOESY analyses were carried out using optimization and spectrum calculation. Conformational searches were carried out by means of the MM+ method in Hyper Chem 8.0 software (Hyperchem Release 8.0, Hypercube, Inc., Gainesville, FL, USA). The lowest energy conformers within 2 kcal/mol were subjected to further DFT calculations. The geometries of the conformers were optimized at the B3LYP/6-31+G(d,p) level in the gas phase using the Gaussian 09 program (Gaussian Inc., Wallingford, CT, USA). The theoretical calculation of ECD was conducted with IEFPCM solvent model for CH_3_OH using TDDFT at the B3LYP/6-31+G(d,p) level for all conformers of compounds (see Supplementary Materials). Boltzmann-weighted ECD spectra was obtained using SpecDis1.70.1 software (University of Würzburg, Würzburg, Germany).

### 3.6. Preparation of Spleen Lymphocytes

Female BALB/c mice (6–8 weeks old, 20 ± 2 g) were purchased from the Department of Laboratory Animal Science (Hainan Medicinal University, China). The mice were sacrificed by cervical dislocation, and their spleens were collected in complete RPMI 1640 medium, which was minced with surgical scissors in a germ-free condition. The suspension was then filtered through a sterile sieve mesh to obtain the single cell suspension. After centrifugation (1000 rpm at 4 °C for 5 min), the resulting cells were treated with erythrocytes lysis buffer, followed by washing twice with cold phosphate-buffered saline (PBS). Then, the cells were adjusted to the concentration of 5 × 10^6^ cells/mL and resuspended in RPMI 1640 medium supplemented with 10% heat-inactivated fetal bovine serum (FBS).

### 3.7. Cell Viability Assay

Cell viability of compounds **1**–**8** were measured using the tetrazolium salt-based CCK-8 assay according to a previously described protocol with some modifications [23]. Spleen lymphocytes were seeded into 96-well plates at a density of 2 × 10^6^ cells/mL onto 96-well plates containing 100 μL of RPMI1640 complete medium (triplicate wells). Then, the cells were treated with 100 μL of various concentrations (3 μg/mL, 6 μg/mL, 12 μg/mL, 25 μg/mL, 50 μg/mL) of isolated compounds or cyclosporine (CsA) at 37 °C with a 5% CO_2_ incubator for 44 h. At the end of the culture, the cell culture plate was removed and observed under an inverted microscope followed by 20 μL of CCK-8 reagent (5 mg/mL). The cells were further incubated for 4 h at 37 °C with 5% CO_2_. The optical density was measured at 450 nm on a microplate reader.

### 3.8. Immunosuppressive Assay

Compounds **1**–**8** were evaluated for immunosuppressive activity against the proliferation of concanavalin A (ConA)-induced T and lipopolysaccharide (LPS)-induced B murine splenic lymphocyte in vitro using a CCK-8 method according to previously reported methods [24]. Cyclosporine A was used as a positive control.

## Figures and Tables

**Table 1:** ^1^H (400 MHz) and ^13^C (125 MHz) NMR spectroscopic data for **1**–**4** in CD_3_OD.

Position	1		2/3		4	
	*δ*_C_, Type	*δ*_H_ (*J* in Hz)	*δ*_C_, Type	*δ*_H_ (*J* in Hz)	*δ*_C_, Type	*δ*_H_ (*J* in Hz)
2	164.8, C		164.8, C		166.7, C	
3	119.1, C		118.2, C		116.8, C	
4	178.4, C		178.9, C		176.8, C	
4a	135.9, C		115.4, C		116.5, C	
5	114.5, C		123.2, C		123.0, C	
6	114.6, CH	6.92, d, 2.4	110.0, CH	7.07, s	110.3, CH	7.09, s
7	165.1, C		151.4, C		152.6, C	
8	102.3, CH	7.10, d, 2.4	138.2, C		140.3, C	
8a	159.5, C		147.1, C		147.2, C	
9	10.3, CH_3_	2.02, s	10.2, CH_3_	2.02, s	96.9, CH	5.73, s
10	171.4, C		172.4, C		35.2, CH_2_	H_a_ 2.77, dd, 17.9, 3.7 H_b_ 2.68, dd, 17.9, 10.7
11	53.5, CH_3_	3.92, s	53.5, CH_3_	3.89, s	63.9, CH	4.40, m
12	57.0, CH_3_	3.92, s	57.2, CH_3_	3.96, s	20.8, CH_3_	1.37, d, 6.2
13					172.1, C	
14					53.3, CH_3_	3.87, s
15					57.2, CH_3_	3.96, s
1′	42.6, CH_2_	H_a_ 2.92, dd, 14.0, 8.0 H_b_ 2.83, dd, 14.0, 5.1	42.4, CH_2_	H_a_ 2.95, dd, 14.1, 7.9 H_b_ 2.83, dd, 14.1, 5.1	18.8, CH_3_	1.19, d, 6.0
2′	67.1, CH	4.25, m	67.1, CH	4.29, m	83.4, CH	3.60, m
3′	23.7, CH_3_	1.29, d, 6.3	23.6, CH_3_	1.29, d, 6.3	73.4, CH	3.54, m
4′					18.3, CH_3_	1.13, d, 6.0

**Table 2:** Immunosuppressive activities of isolated compounds **1**–**8.**

Compound	Cytotoxicity ^a^ IC_50_ (μM) ^b^	ConA-Induced T-Cell Proliferation	LPS-Induced B-Cell Proliferation
		IC_50_ (μM) ^b^	IC_50_ (μM) ^b^
**5**	47	34	117
**1**–**4, 6**–**8**	-	-	-
**CsA** ^c^	11	4	25

^a^ Cell viability on murine splenocytes was tested by using CCK-8 method. ^b^ Data are presented as mean ± SD (n = 3) in μM**.** ^c^ Positive control.

## Data Availability

Data is contained within the article or Supplementary Materials.

## Supplementary Materials

The following are available online at https://www.mdpi.com/article/10.3390/md19060348/s1. Figures S1–S36: Copies of HR-ESI-MS, 1D- and 2D-NMR spectra of **1**–**4**. Tables S1–S4: Gibbs free energies^a^ and equilibrium populations^b^ of low-energy conformers of **1** and **4**; Cartesian coordinates for the low-energy reoptimized MMFF conformers of **1** and **4** at B3LYP/6-31G(d,p) level of theory in gas.
