# Supplementary material for: Four New Chromones from the Endophytic Fungus Phomopsis asparagi DHS-48 Isolated from the Chinese Mangrove Plant Rhizophora mangle
Source: Mar Drugs. 2021 Jun 19;19(6):348. doi: 10.3390/md19060348 (PMC8235223; doi:10.3390/md19060348)
Supplement: Supplementary file 1 [file marinedrugs-19-00348-s001.zip › marinedrugs-1269640-SI.pdf]

Supporting Information

Four New Chromones from the Endophytic Fungus *Phomopsis asparagi* DHS-48 Isolated from the Chinese Mangrove Plant *Rhizophora mangle*

Chengwen Wei,[a] Chunxiao Sun,[b] Zhao Feng,[a] Xuexia Zhang,[a] Jing Xu [a,*]

[a] School of Chemical Engineering and Technology, Hainan University, Haikou 570228, P. R. China.

[b] School of Medicine and Pharmacy, Ocean University of China, Qingdao 266003, P. R. China.

[*] To whom correspondence should be addressed.

Prof. Dr. Jing Xu, Tel.: ++86–898–6627–9226, Fax: ++86–898–6627–9010, E–mail: *happyjing3@163.com*

Contents

Figure S1. ^{1}H-NMR of phomochromenone D (**1**) -------------------------------------- 4

Figure S2. ^{13}C-NMR of phomochromenone D (**1**)------------------------------------4

Figure S3. DEPT of phomochromenone D (**1**)--5

Figure S4. ^{1}H-^{1}H COSY of phomochromenone D (**1**) --------------------------------- 5

Figure S5. HMQC of phomochromenone D (**1**)-- 6

Figure S6. HMBC of phomochromenone D (**1**)---------------------------------------6

Figure S7. NOESY of phomochromenone D (**1**)--7

Figure S8. HR-ESI-MS of phomochromenone D (**1**)-------------------------------------7

Figure S9. ^{1}H-NMR of phomochromenone E (**2**) and F (**3**) ----------------------------- 7

Figure S10. ^{13}C-NMR of phomochromenone E (**2**) and F (**3**) ---------------------------8

Figure S11. DEPT of phomochromenone E (**2**) and F (**3**) --------------------------------8

Figure S12. ^{1}H-^{1}H COSY of phomochromenone E (**2**) and F (**3**) ---------------------- 9

Figure S13. HMQC of phomochromenone E (**2**) and F (**3**) ----------------------------- 9

Figure S14. HMBC of phomochromenone E (**2**) and F (**3**) -----------------------------10

Figure S15. HR-ESI-MS of phomochromenone E (**2**) and F (**3**) ----------------------10

Figure S16. ^{1}H-NMR of phomochromenone G (**4**) -------------------------------------10

Figure S17. ^{13}C-NMR of phomochromenone G (**4**)---------------------------------11

Figure S18. DEPT of phomochromenone G (**4**)--11

Figure S19. ^{1}H-^{1}H COSY of phomochromenone G (**4**)----------------------------------12

Figure S20. HMQC of phomochromenone G (**4**)--12

Figure S21. HMBC of phomochromenone G (**4**)--------------------------------------13

Figure S22. NOESY of phomochromenone G (**4**)--13

Figure S23. HR-ESI-MS of phomochromenone G (**4**)------------------------------------13

Figure S24. UPLC analysis profile of (*R*)- and (*S*)- MPA esters of **2** and **3** ----------14

Figure S25. HR-ESI-MS of (*R*)-MPA ester **2** --14

Figure S26. ^{1}H-NMR of (*R*)-MPA ester **2** --15

Figure S27. ^{1}H-^{1}H COSY of (*R*)-MPA ester **2**---15

Figure S28. HR-ESI-MS of (*S*)-MPA ester **2**--16

Figure S29. ^{1}H-NMR of (*S*)-MPA ester **2**--16

Figure S30. ^{1}H-^{1}H COSY of (*S*)-MPA ester **2**---17

Figure S31. HR-ESI-MS of (*R*)-MPA ester **3** --17
Figure S32. ^{1}H-NMR of (*R*)-MPA ester **3**---18
Figure S33. ^{1}H-^{1}H COSY of (*R*)-MPA ester **3**--18
Figure S34. HR-ESI-MS of (*S*)-MPA ester **3**--19
Figure S35. ^{1}H-NMR of (*S*)-MPA ester **3**---19
Figure S36. ^{1}H-^{1}H COSY of (*S*)-MPA ester **3**---20
Table S1. Gibbs free energies^a and equilibrium populations^b of low-energy conformers of phomochromenone D (**1**)-- 20
Table S2. Cartesian coordinates for the low-energy reoptimized MMFF conformers of phomochromenone D (**1**) at B3LYP/6-31G(d,p) level of theory in gas----------------20
Table S3. Gibbs free energies^a and equilibrium populations^b of low-energy conformers of phomochromenone G (**4**)-- 26
Table S4. Cartesian coordinates for the low-energy reoptimized MMFF conformers of phomochromenone G (**4**) at B3LYP/6-31G(d,p) level of theory in gas----------------26

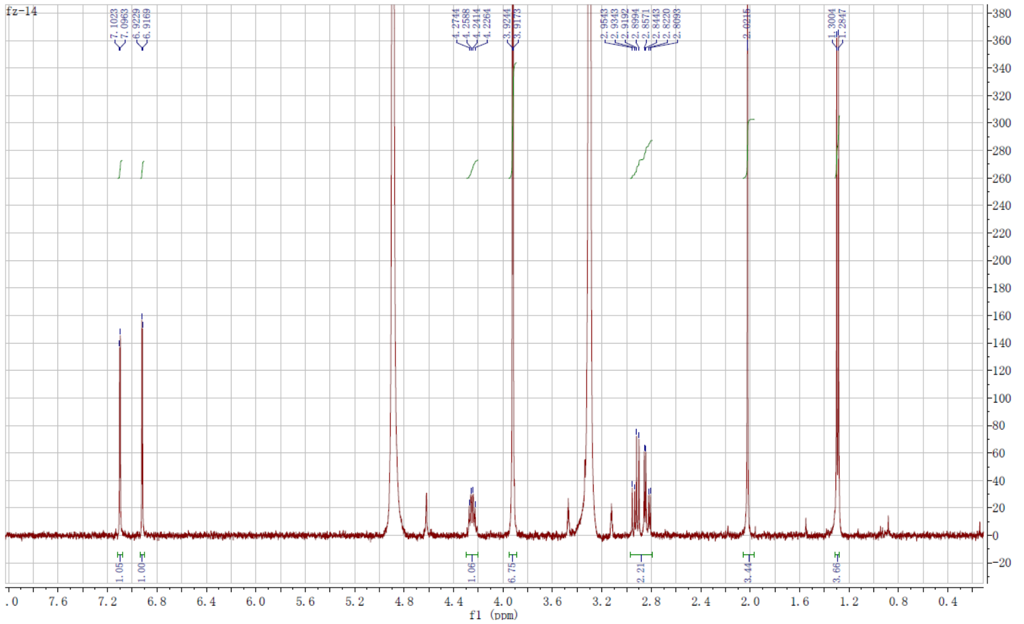

Figure S1. ^{1}H-NMR of phomochromenone D (**1**)

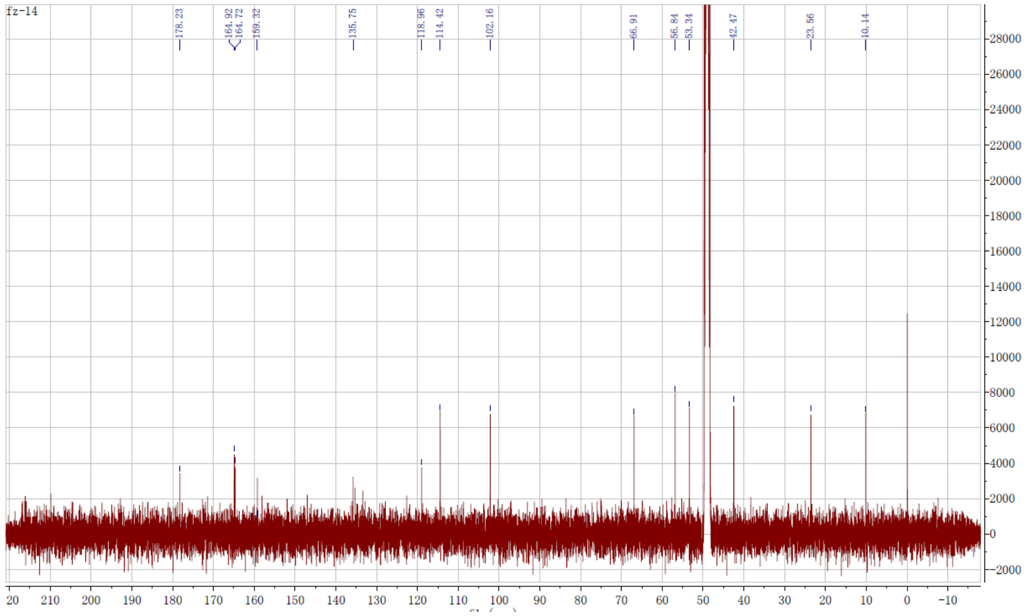

Figure S2. ^{13}C-NMR of phomochromenone D (**1**)

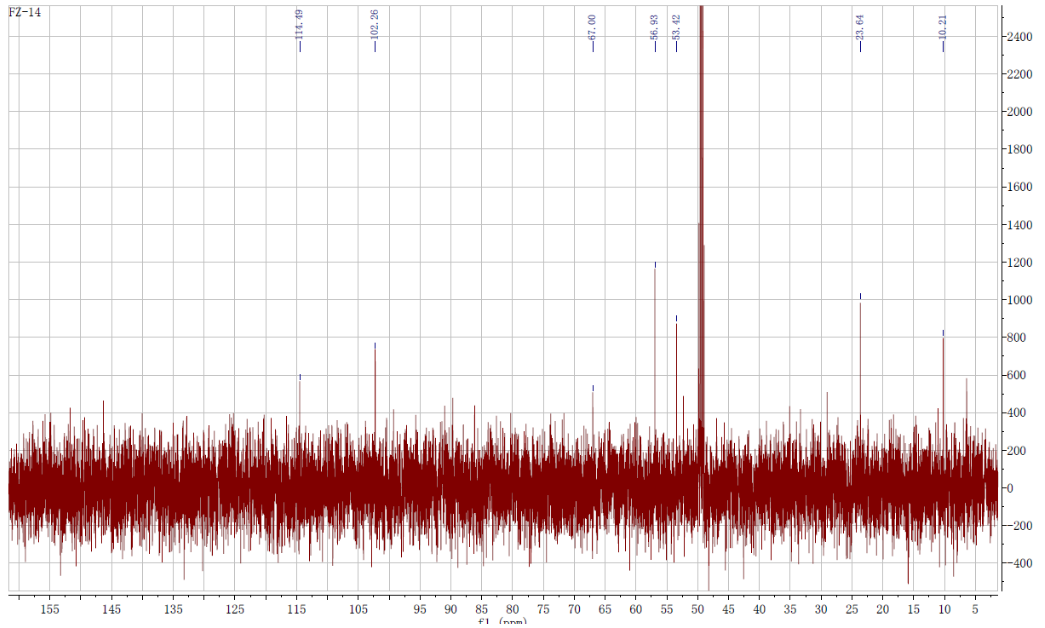

Figure S3. DEPT of phomochromenone D (**1**)

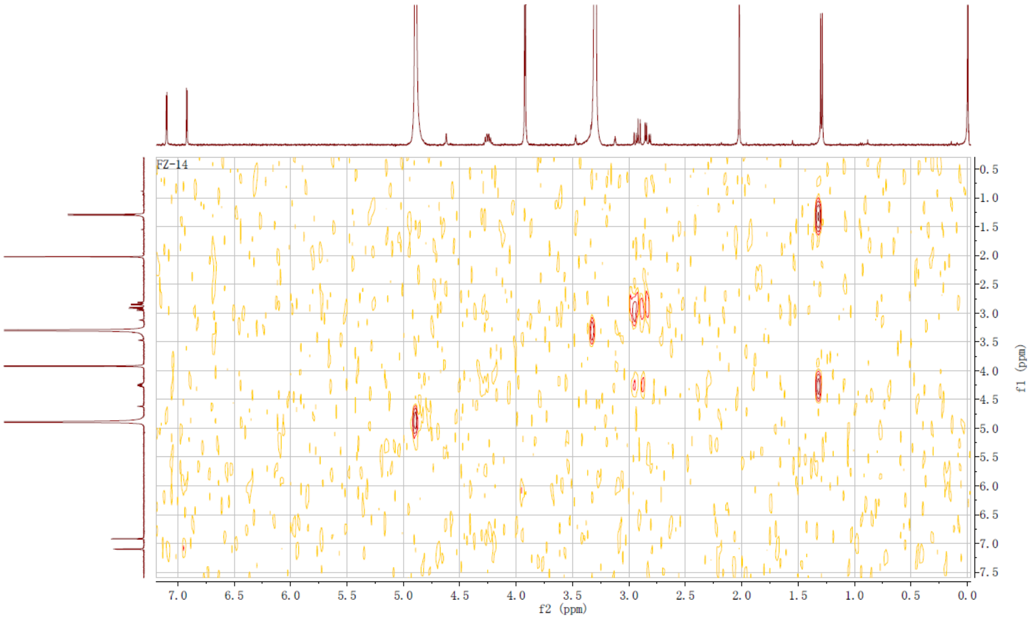

Figure S4. ^{1}H-^{1}H COSY of phomochromenone D (**1**)

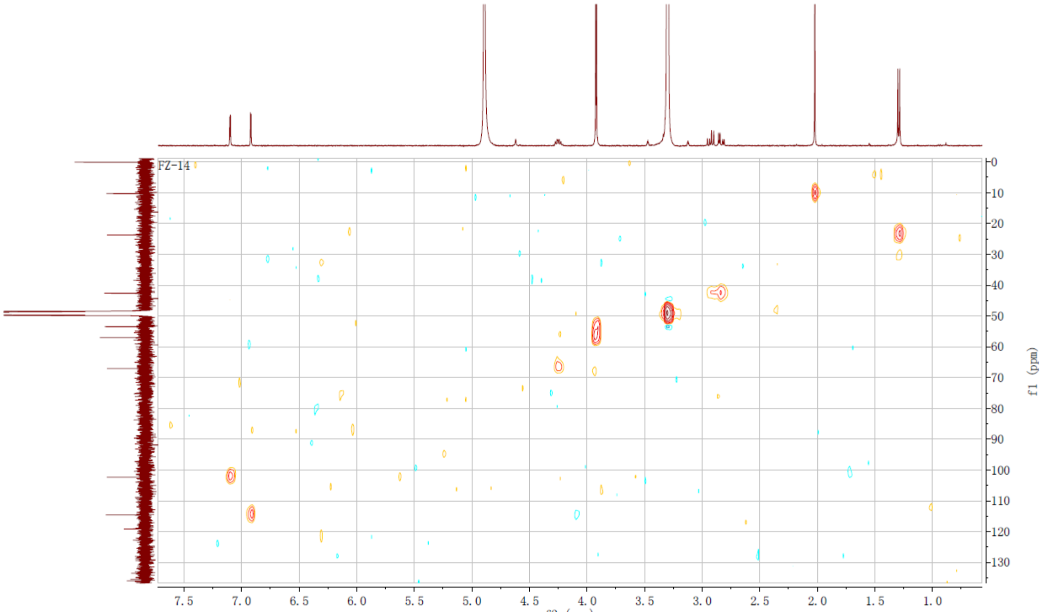

Figure S5. HMQC of phomochromenone D (**1**)

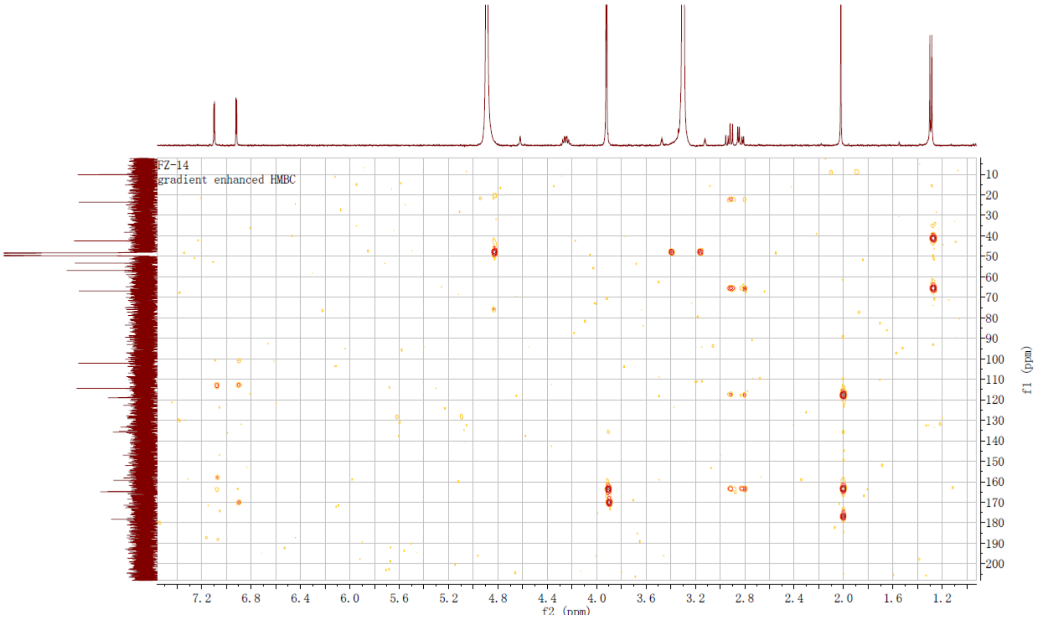

Figure S6. HMBC of phomochromenone D (**1**)

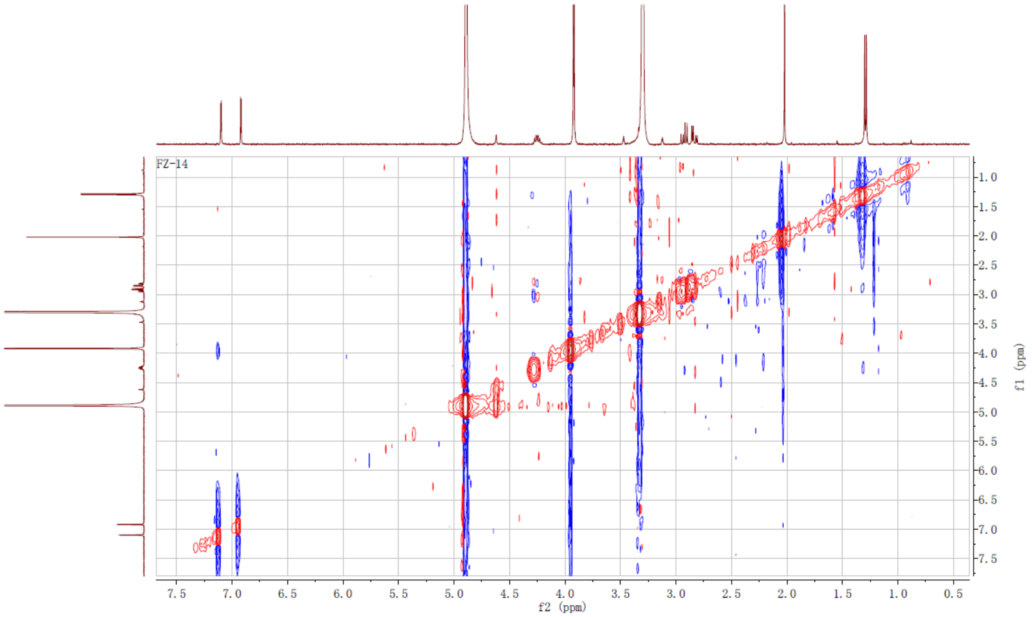

Figure S7. NOESY of phomochromenone D (**1**)

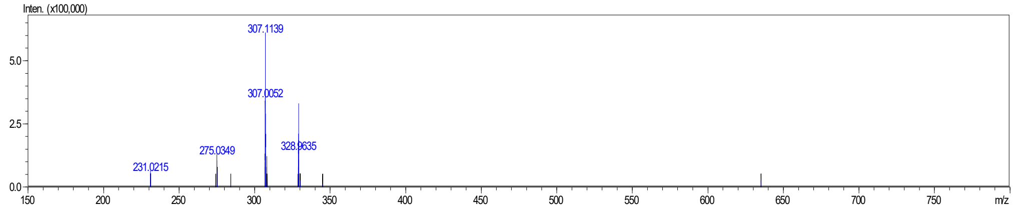

Figure S8. HR-ESI-MS of phomochromenone D (**1**)

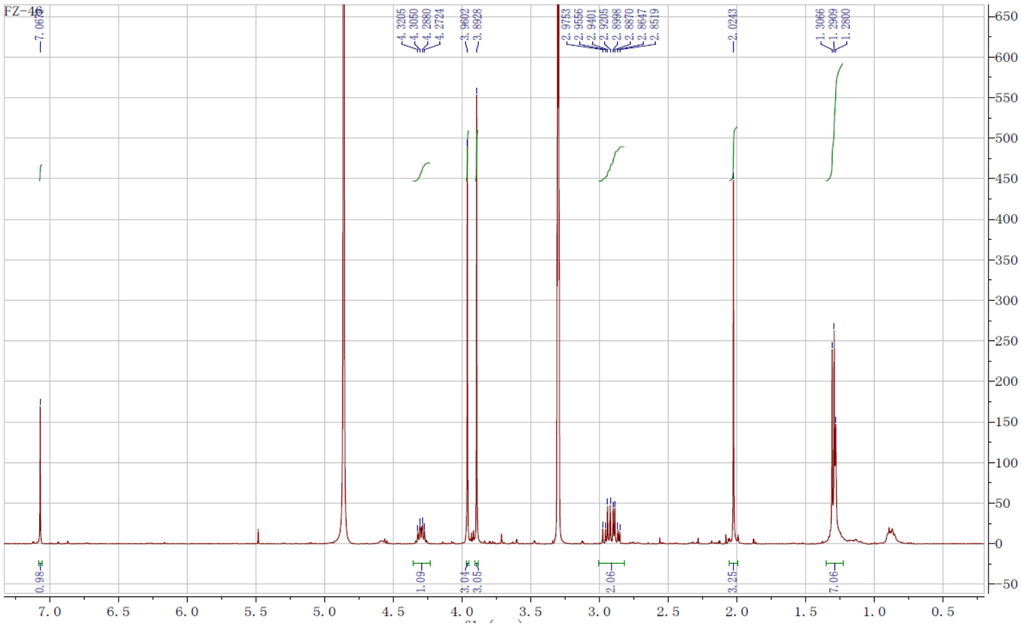

Figure S9. ^{1}H-NMR of phomochromenones E (**2**) and F (**3**)

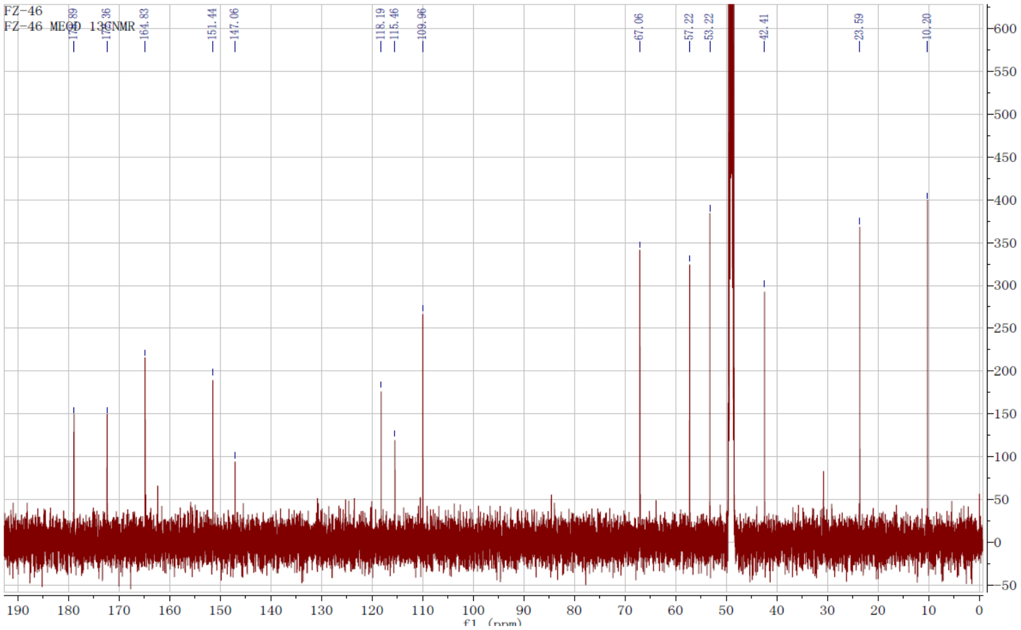

Figure S10. ^{13}C-NMR of phomochromenones E (**2**) and F (**3**)

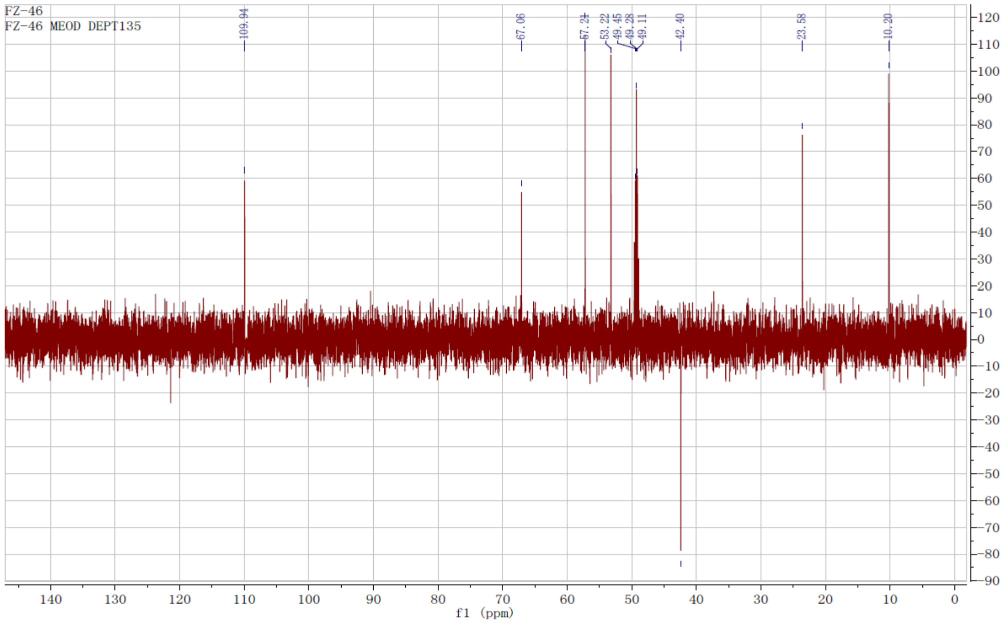

Figure S11. DEPT of phomochromenones E (**2**) and F (**3**)

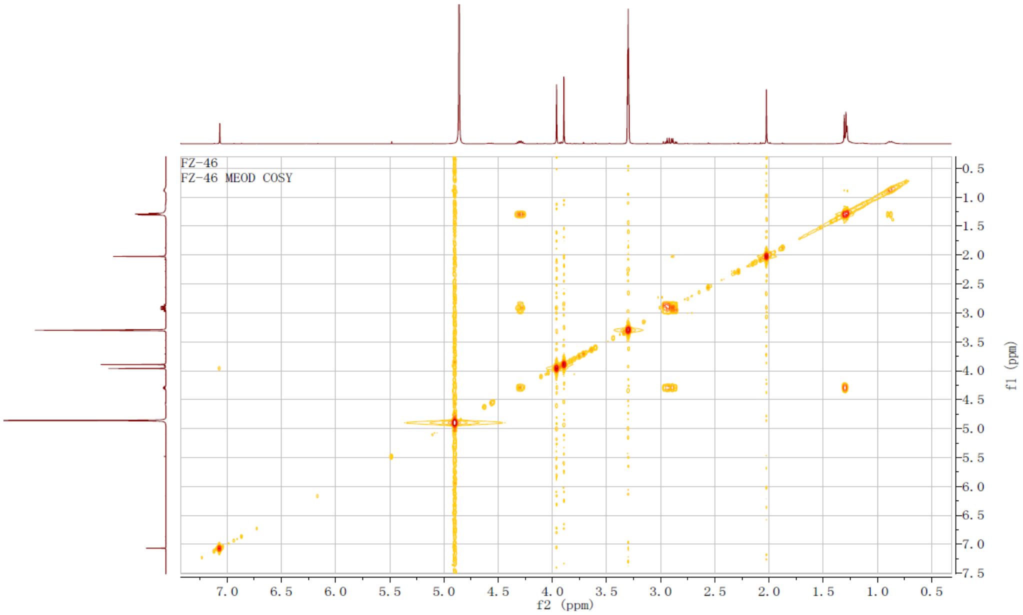

Figure S12. ^{1}H-^{1}H COSY of phomochromenones E (**2**) and F (**3**)

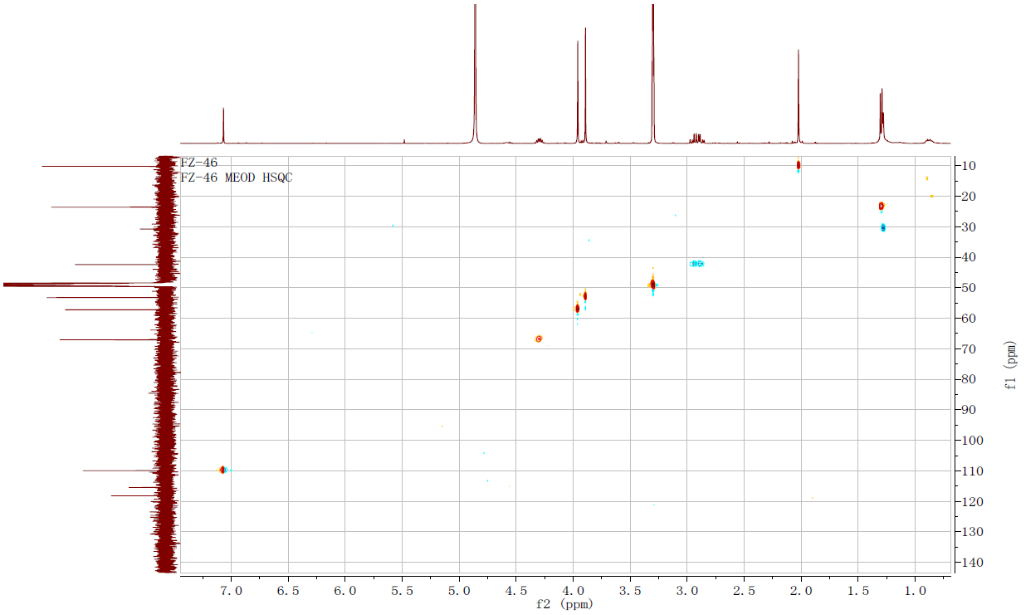

Figure S13. HMQC of phomochromenones E (**2**) and F (**3**)

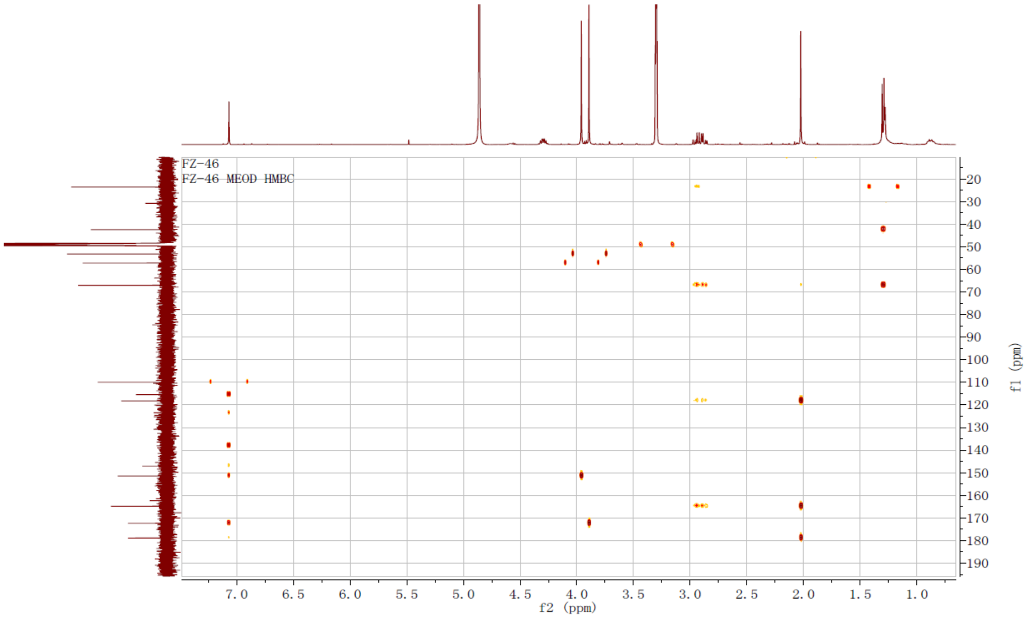

Figure S14. HMBC of phomochromenones E (**2**) and F (**3**)

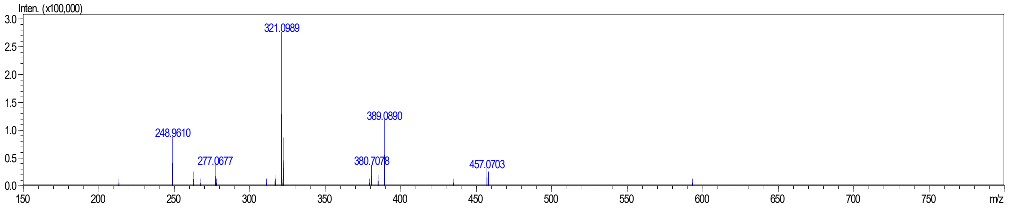

Figure S15. HR-ESI-MS of phomochromenones E (**2**) and F (**3**)

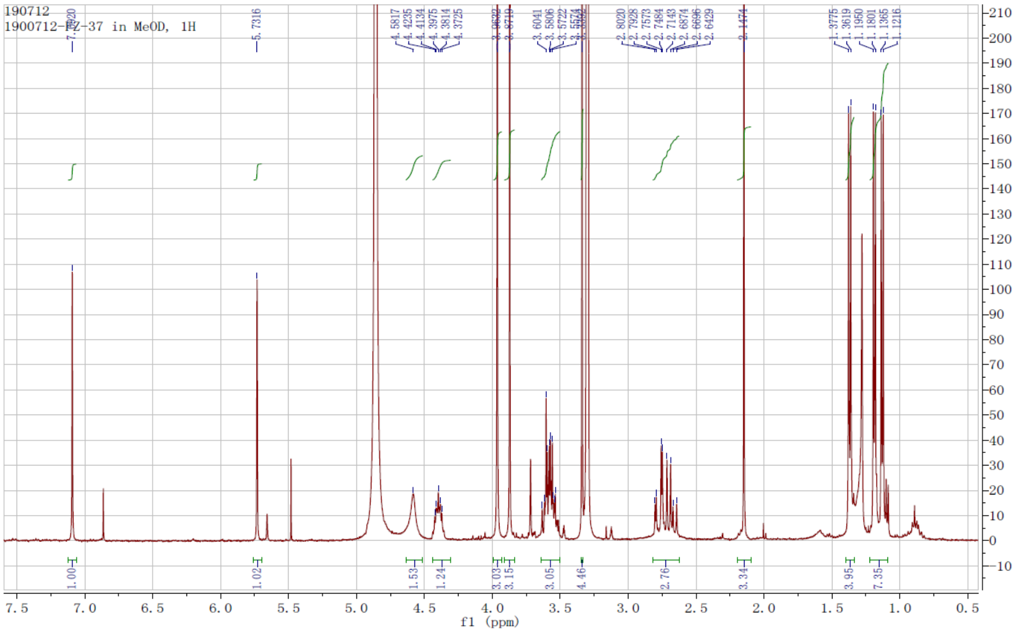

Figure S16. ^{1}H-NMR of phomochromenone G (**4**)

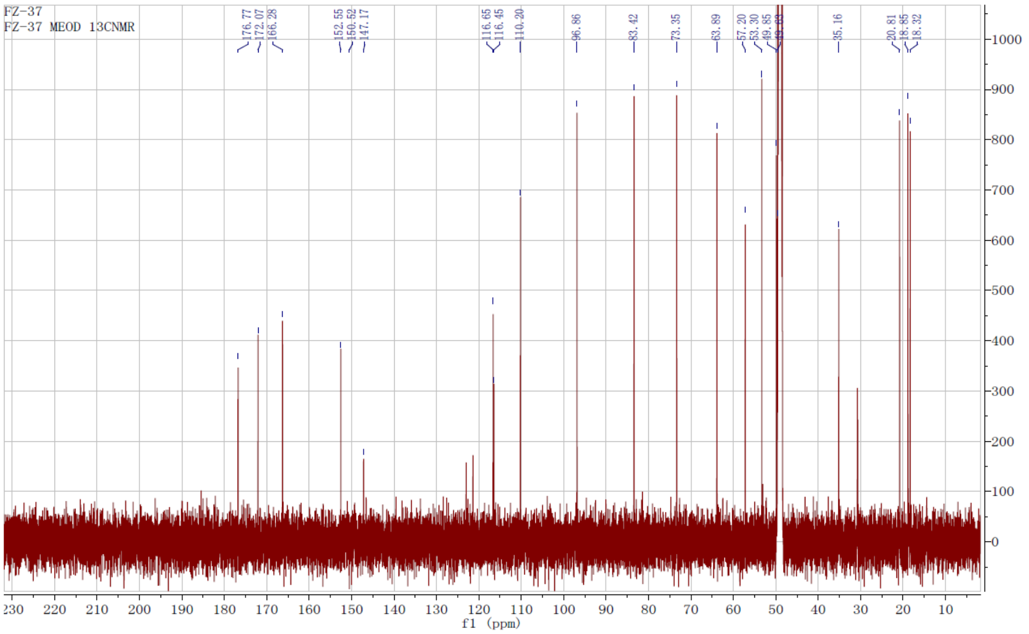

Figure S17. ^{13}C-NMR of phomochromenone G (**4**)

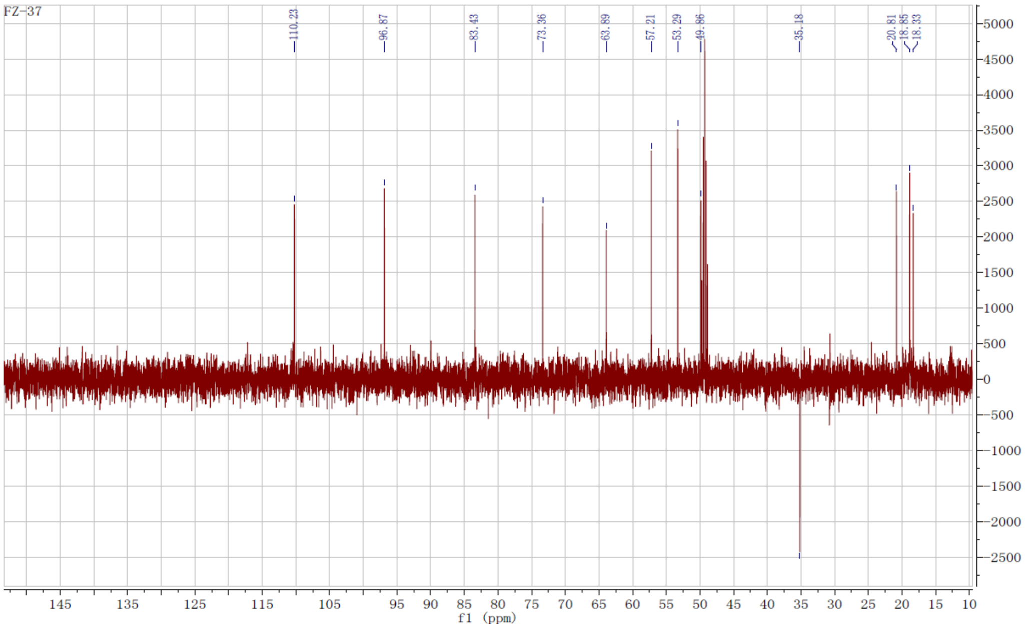

Figure S18. DEPT of phomochromenone G (**4**)

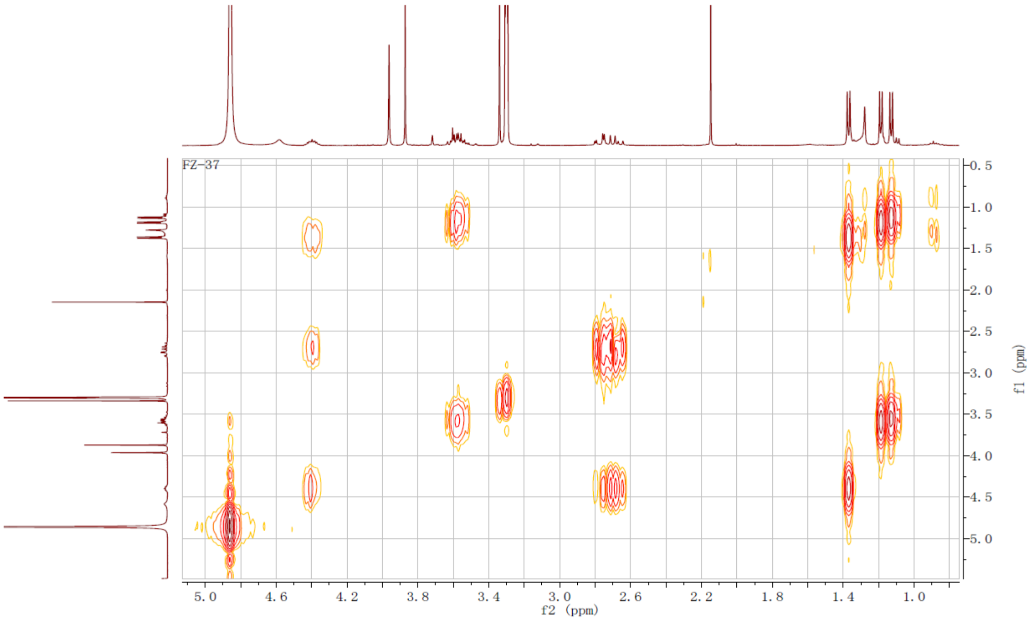

Figure S19. ^{1}H-^{1}H COSY of phomochromenone G (**4**)

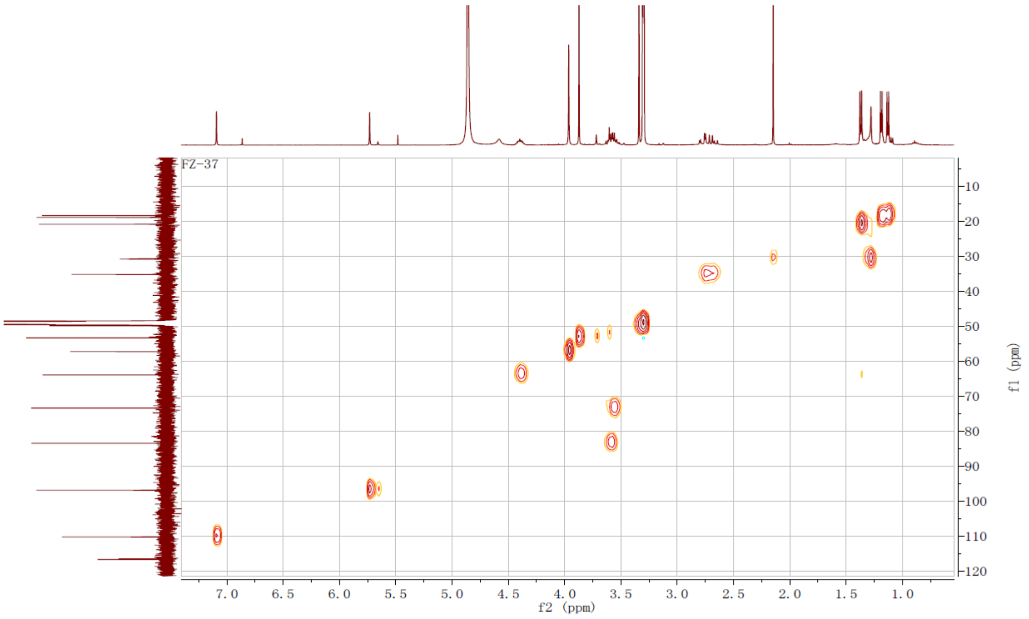

Figure S20. HMQC of phomochromenone G (**4**)

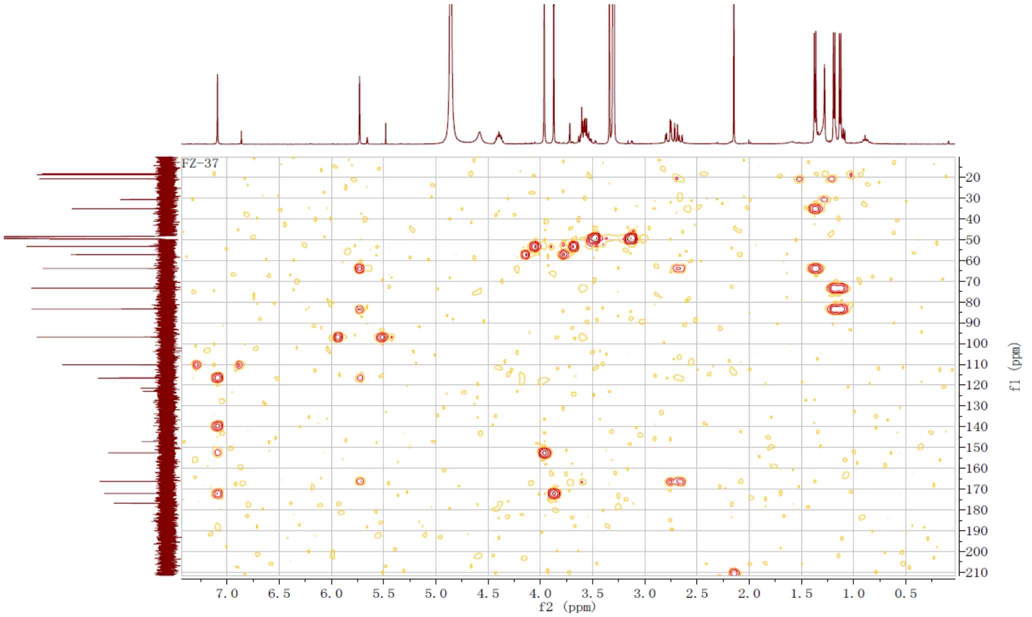

Figure S21. HMBC of phomochromenone G (**4**)

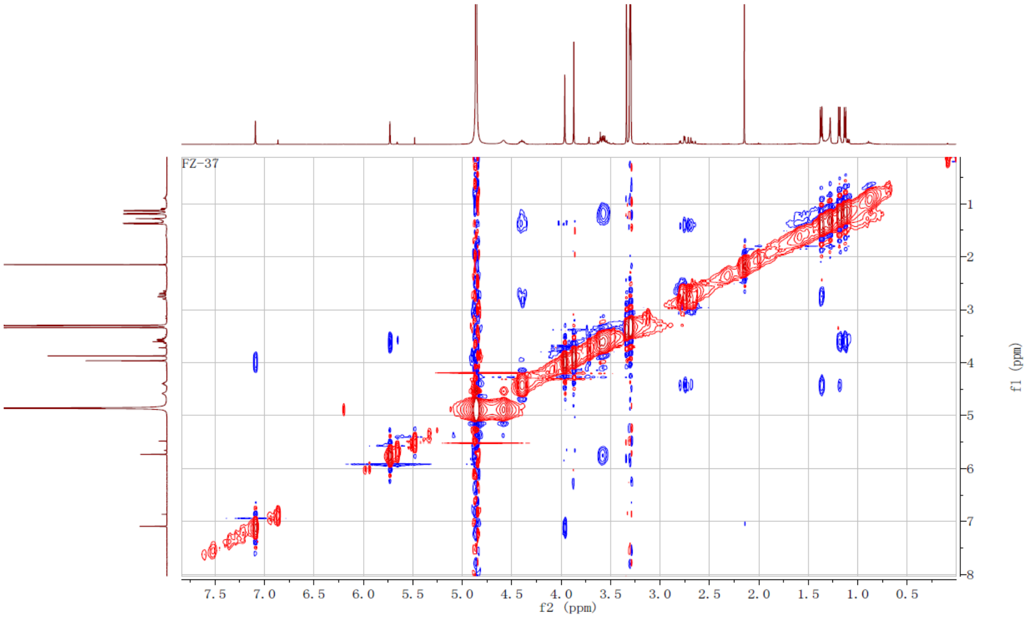

Figure S22. NOESY of phomochromenone G (**4**)

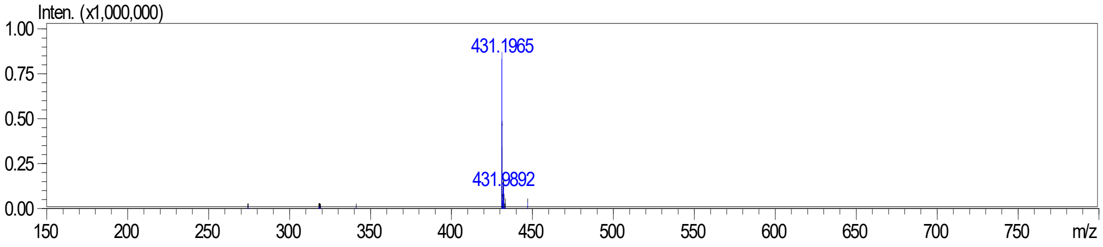

Figure S23. HR-ESI-MS of phomochromenone G (**4**)

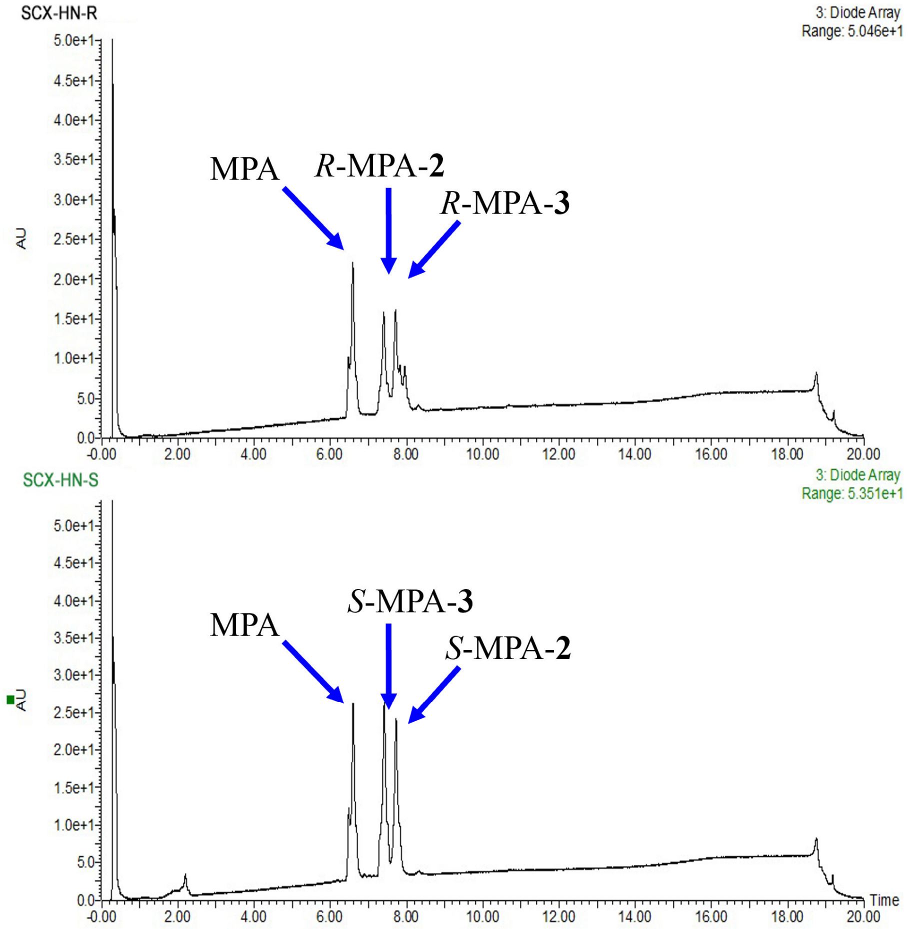

Figure S24. UPLC analysis profile of (*R*)- and (*S*)- MPA esters of **2** and **3**

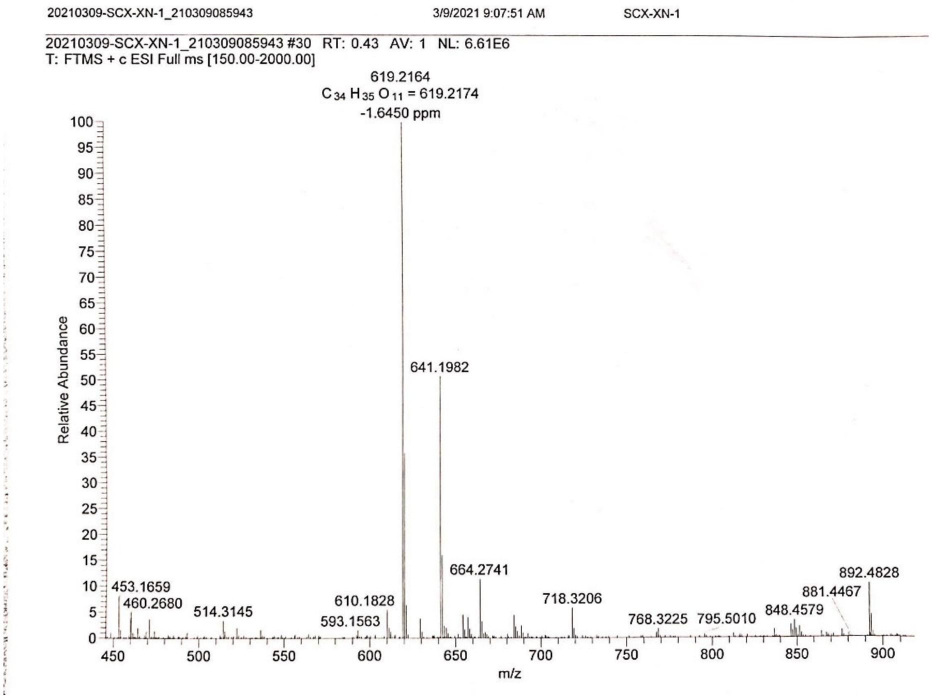

Figure S25. HR-ESI-MS of (*R*)-MPA ester **2**

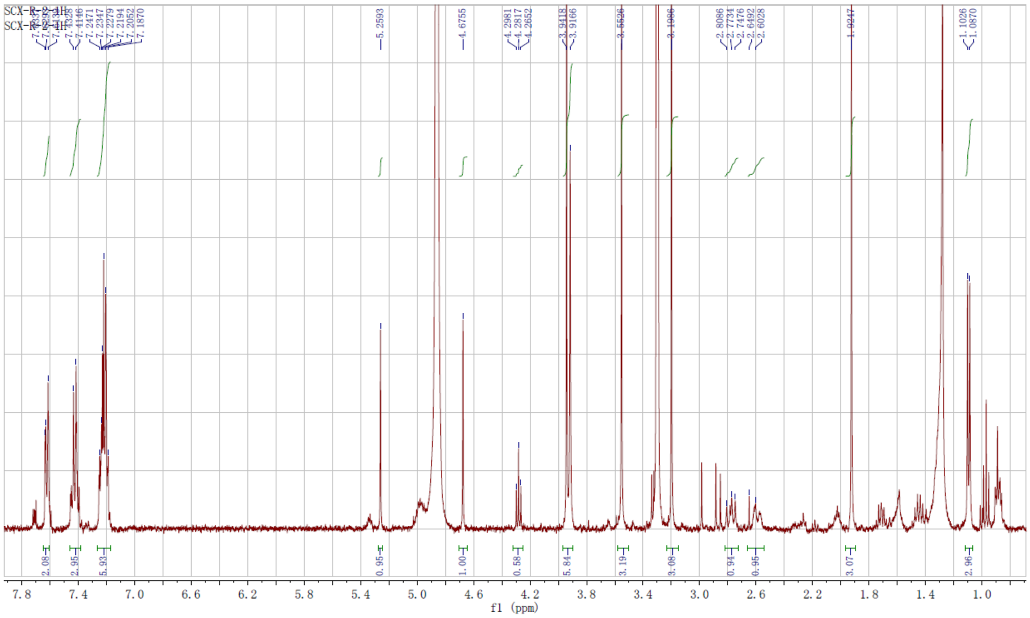

Figure S26. ^{1}H-NMR of (*R*)-MPA ester **2**

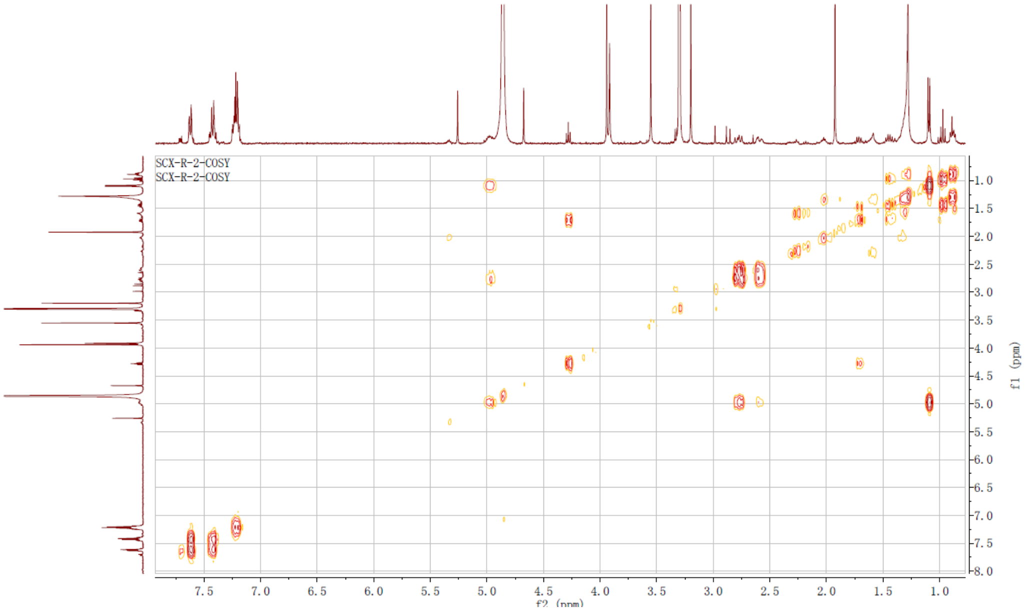

Figure S27. ^{1}H-^{1}H COSY of (*R*)-MPA ester **2**

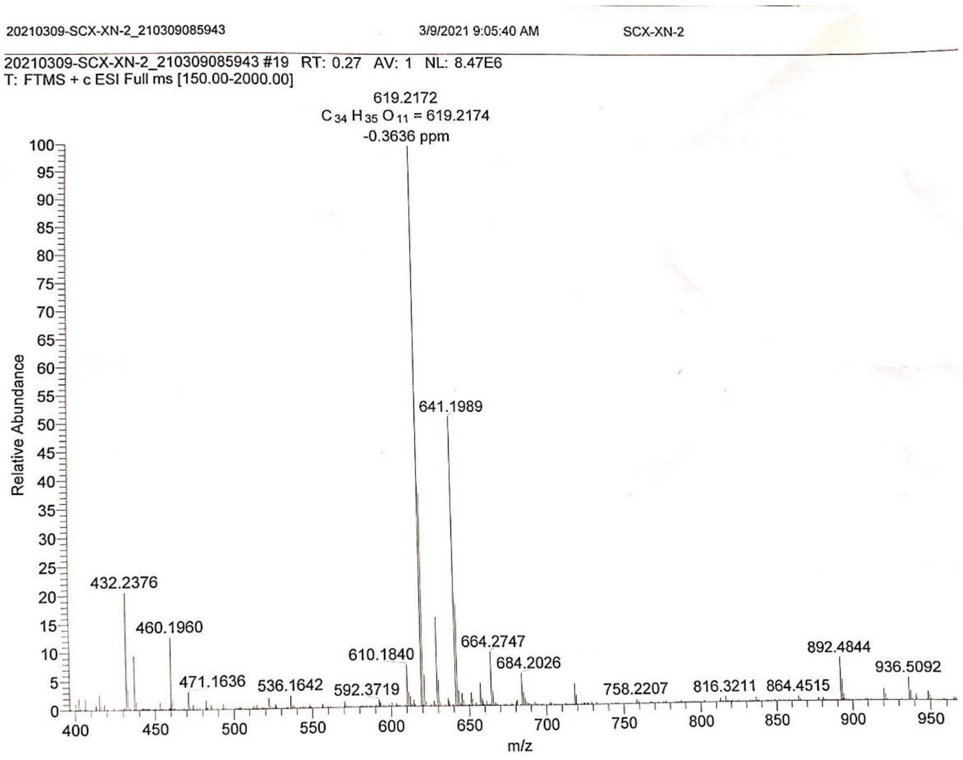

Figure S28. HR-ESI-MS of (*S*)-MPA ester **2**

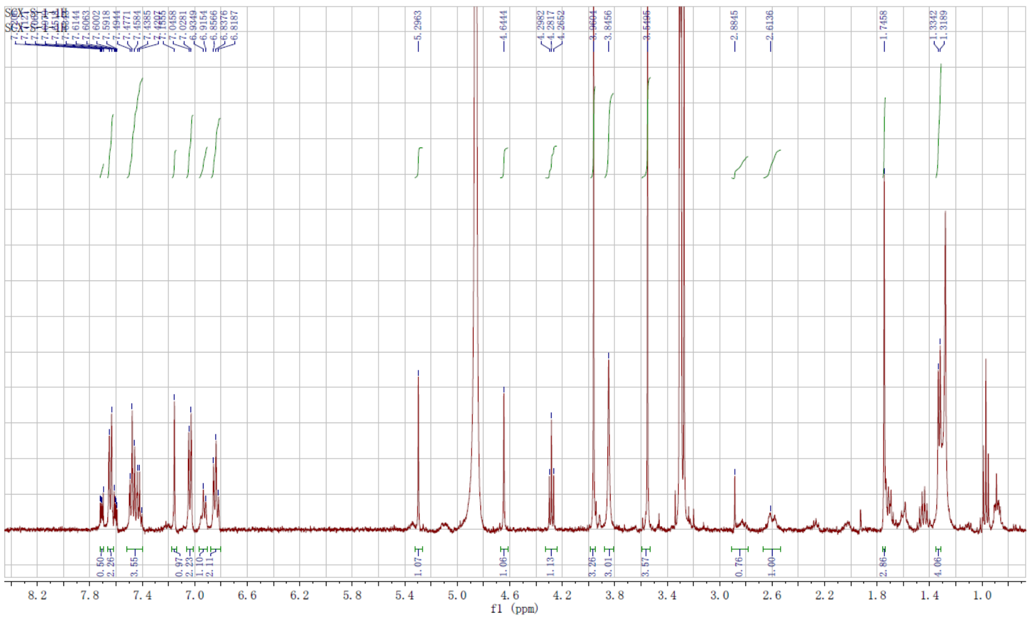

Figure S29. ^{1}H-NMR of (*S*)-MPA ester **2**

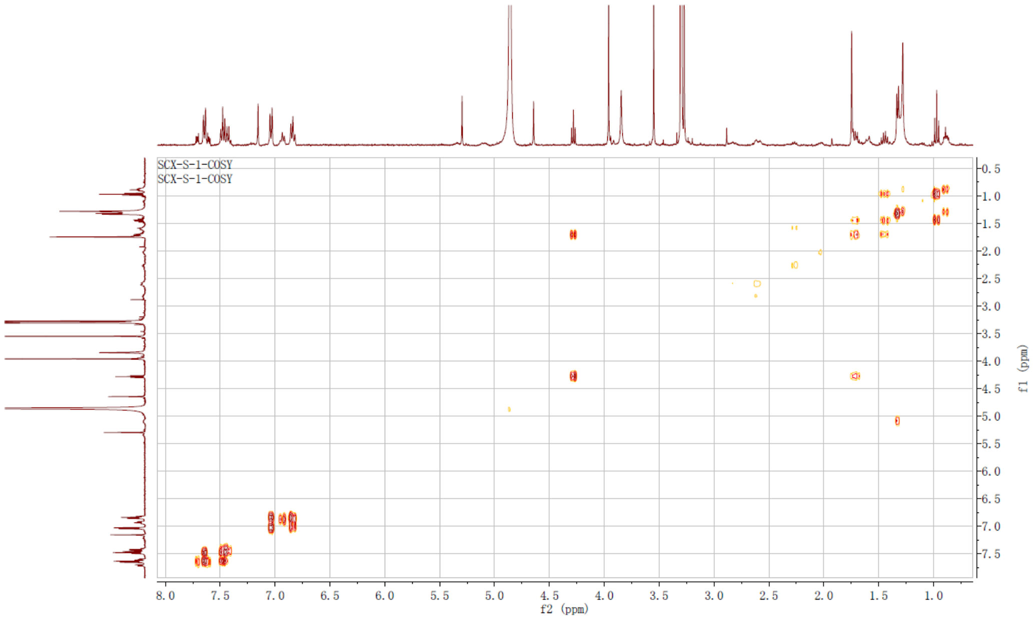

Figure S30. ^{1}H-^{1}H COSY of (*S*)-MPA ester **2**

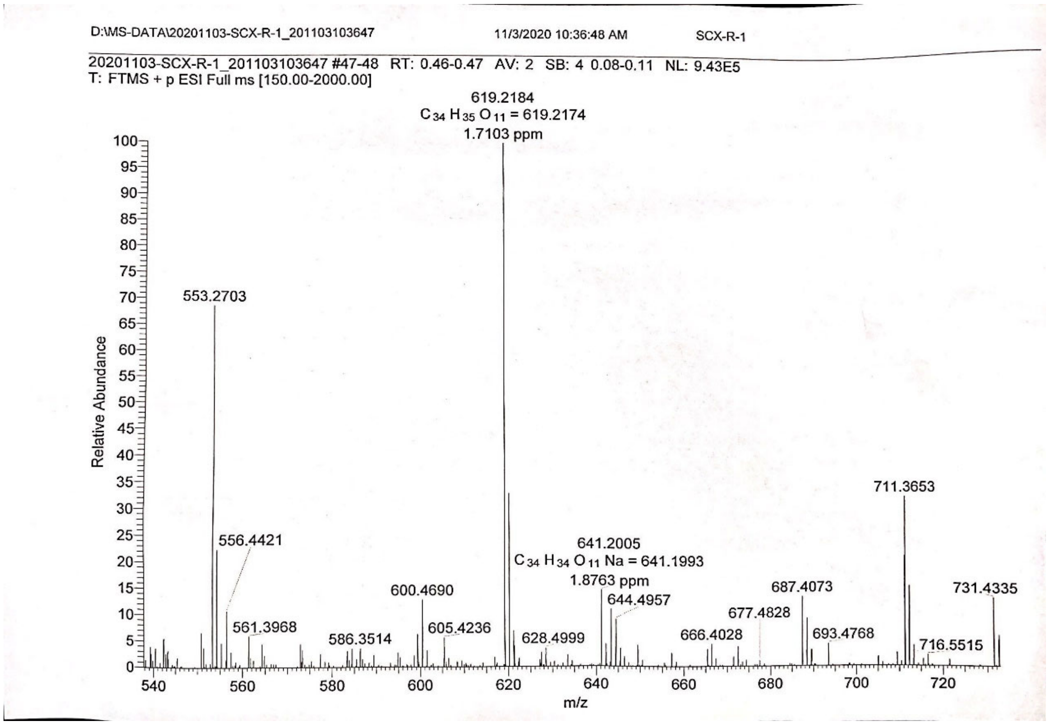

Figure S31. HR-ESI-MS of (*R*)-MPA ester **3**

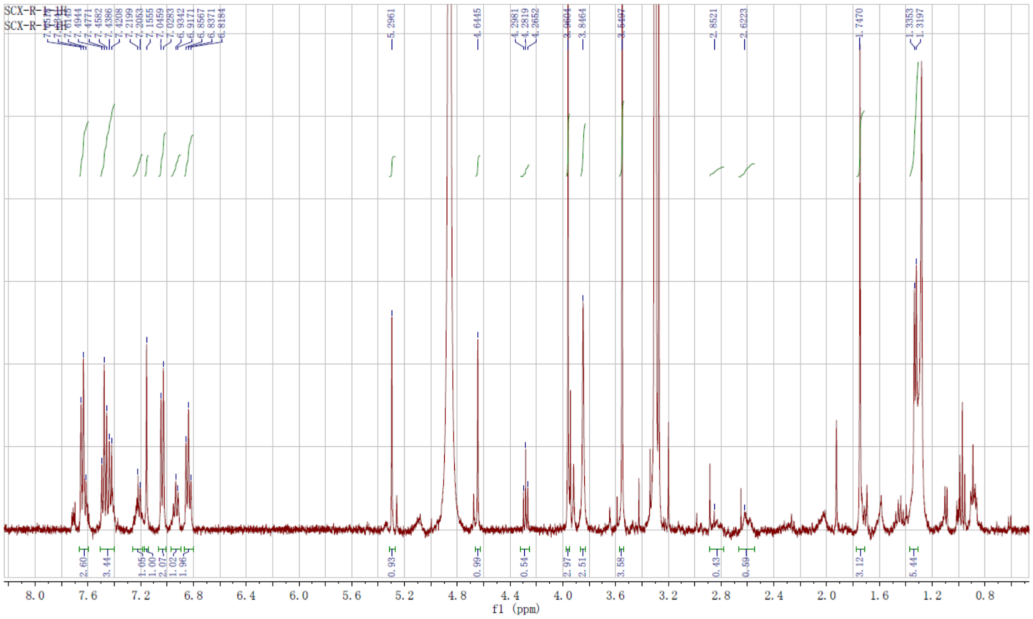

Figure S32. ¹H-NMR of (*R*)-MPA ester **3**

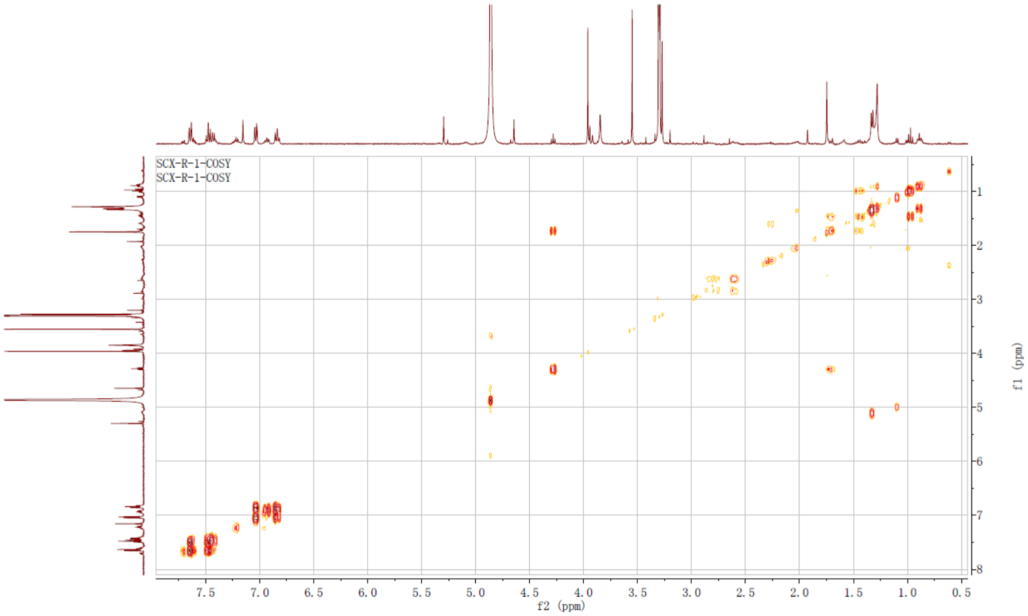

Figure S33. ^{1}H-^{1}H COSY of (*R*)-MPA ester **3**

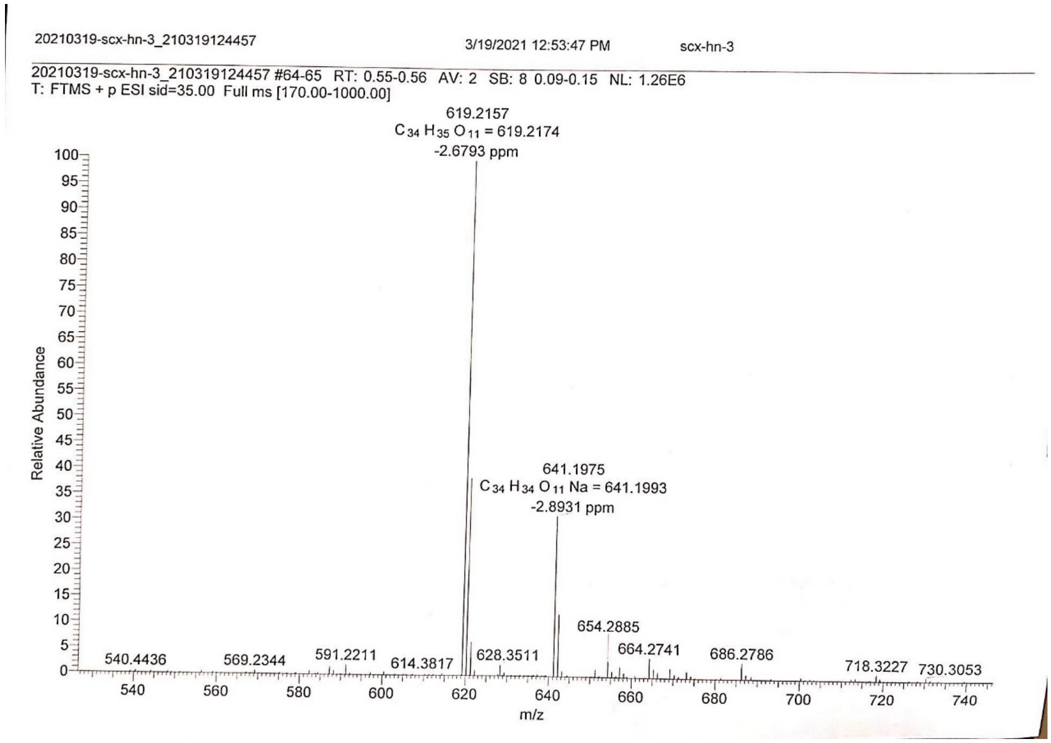

Figure S34. HR-ESI-MS of (*S*)-MPA ester **3**

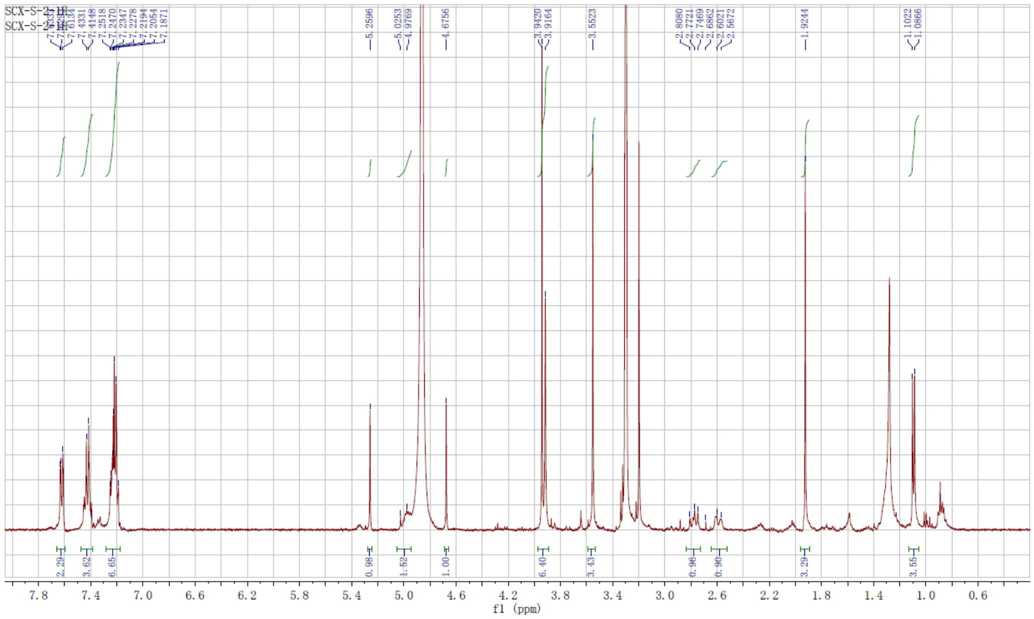

Figure S35. ^{1}H-NMR of (*S*)-MPA ester **3**

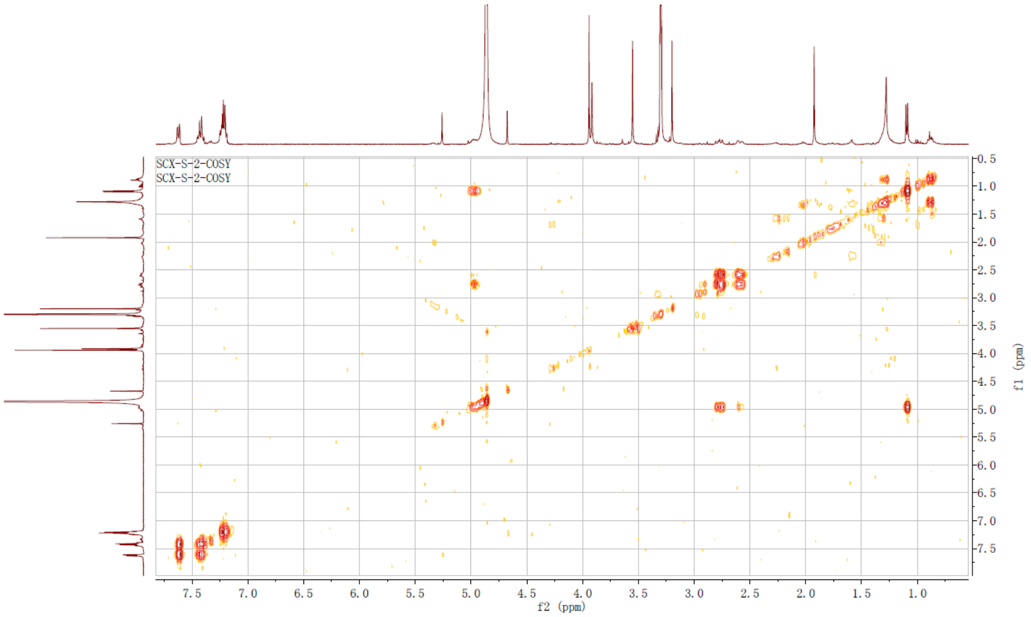

Figure S36. ^{1}H-^{1}H COSY of (*S*)-MPA ester **3**

Table S1..Gibbs free energies^a and equilibrium populations^b of low-energy conformers of phomochromenone D (**1**).

Conformers	In gas	
	G^a	P (%)^b
**1.-1**	-672458.42332812	9.81
**1.-2**	-672458.48607912	10.90
**1.-3**	-672458.92847367	23.02
**1-4**	-672459.28552686	42.08
**1-5**	-672458.6417016	14.18

[a]B3LYP/6-31G(d,p), in kcal/mol. [b]From *G* values at 298.15K.

Table S2. Cartesian coordinates for the low-energy reoptimized MMFF conformers of phomochromenone D (**1**) at B3LYP/6-31G(d,p) level of theory in gas

Conformer **1-1**

**1-1**		Standard Orientation (Ångstroms)			
Center number	Atomic number	Atomic Type	X	Y	Z
1.	6.	0.	-0.491833	3.153694	-0.217427
2.	6.	0.	-0.505483	2.877390	1.163643
3.	6.	0.	-0.272400	1.580240	1.621524
4.	6.	0.	-0.023569	0.578458	0.689750
5.	6.	0.	0.022668	0.823512	-0.692490
6.	6.	0.	-0.225901	2.139556	-1.133144
7.	8.	0.	0.182271	-0.676358	1.188370
8.	6.	0.	0.404055	-1.732413	0.343672
9.	6.	0.	0.463344	-1.608616	-1.005926
10.	6.	0.	0.310854	-0.281284	-1.619454
11.	6.	0.	0.559449	-2.995344	1.139508
12.	6.	0.	-0.777584	-3.564109	1.644978
13.	8.	0.	-1.510172	-3.942434	0.480235
14.	6.	0.	0.689263	-2.784687	-1.923254
15.	8.	0.	0.425792	-0.090794	-2.835528
16.	6.	0.	-0.355194	2.512472	-2.600096
17.	8.	0.	0.747322	2.887438	-3.281738
18.	8.	0.	-1.446119	2.634433	-3.102799
19.	8.	0.	-0.745239	3.805988	2.123992
20.	6.	0.	-1.027088	5.144660	1.728466
21.	6.	0.	2.055211	2.597843	-2.774806
22.	6.	0.	-0.569321	-4.753499	2.582392
23.	1.	0.	-0.708346	4.149146	-0.583948
24.	1.	0.	-0.295491	1.358635	2.681481
25.	1.	0.	1.054097	-3.758463	0.534049
26.	1.	0.	1.198352	-2.781796	2.003699
27.	1.	0.	-1.308980	-2.764608	2.184862
28.	1.	0.	-2.398536	-4.205773	0.752877
29.	1.	0.	1.712577	-3.171692	-1.846537
30.	1.	0.	0.537076	-2.455366	-2.952214
31.	1.	0.	-0.003167	-3.599077	-1.698489
32.	1.	0.	-1.192261	5.697516	2.653588
33.	1.	0.	-1.928569	5.198943	1.107213
34.	1.	0.	-0.184480	5.590076	1.186349

35.	1.	0.	2.252651	1.526300	-2.844908
36.	1.	0.	2.175137	2.937678	-1.741381
37.	1.	0.	2.745531	3.146855	-3.416524
38.	1.	0.	-0.016624	-5.549329	2.073067
39.	1.	0.	-1.533131	-5.163079	2.904893
40.	1.	0.	-0.016163	-4.461111	3.481055

Conformer **1-2**

**1-2**		Standard Orientation (Ångstroms)			
Center number	Atom number	Type	X	Y	Z
1.	6.	0.	-0.011457	3.249896	-0.202102
2.	6.	0.	0.008252	2.968237	1.177726
3.	6.	0.	0.223114	1.663860	1.623890
4.	6.	0.	0.405572	0.657896	0.681331
5.	6.	0.	0.367211	0.899030	-0.701917
6.	6.	0.	0.161768	2.226614	-1.129783
7.	8.	0.	0.635561	-0.596758	1.169920
8.	6.	0.	0.837734	-1.648764	0.316147
9.	6.	0.	0.800801	-1.531785	-1.034841
10.	6.	0.	0.541413	-0.218318	-1.642269
11.	6.	0.	1.085315	-2.902743	1.102518
12.	6.	0.	-0.186601	-3.482862	1.743129
13.	8.	0.	-1.035562	-3.864157	0.660453
14.	6.	0.	0.986683	-2.709661	-1.959230
15.	8.	0.	0.452904	-0.053046	-2.864244
16.	6.	0.	0.250997	2.652731	-2.585144
17.	8.	0.	-0.861993	2.628873	-3.347328
18.	8.	0.	1.260366	3.163655	-3.007058
19.	8.	0.	-0.162550	3.901601	2.148126
20.	6.	0.	-0.351288	5.260582	1.766765
21.	6.	0.	-2.014517	1.881711	-2.940840
22.	6.	0.	0.127861	-4.672281	2.650146
23.	1.	0.	-0.139237	4.264669	-0.557105
24.	1.	0.	0.256389	1.443374	2.683816
25.	1.	0.	1.527751	-3.664025	0.456069
26.	1.	0.	1.803965	-2.674462	1.897768
27.	1.	0.	-0.664774	-2.688917	2.338254
28.	1.	0.	-1.870952	-4.179163	1.029293
29.	1.	0.	2.022058	-3.070294	-1.956547

30.	1.	0.	0.749199	-2.391853	-2.975608
31.	1.	0.	0.333115	-3.539433	-1.679526
32.	1.	0.	-0.458254	5.816740	2.698448
33.	1.	0.	-1.258253	5.386805	1.163664
34.	1.	0.	0.511411	5.646085	1.211157
35.	1.	0.	-2.318905	2.126431	-1.918460
36.	1.	0.	-1.814548	0.812020	-3.028592
37.	1.	0.	-2.808180	2.170652	-3.631063
38.	1.	0.	0.624625	-5.465402	2.082404
39.	1.	0.	-0.792977	-5.086149	3.076554
40.	1.	0.	0.774982	-4.378391	3.483080

Conformer **1-3**

**1-3**		Standard Orientation (Ångstroms)			
Center number	Atom number	Type	X	Y	Z
1.	6.	0.	-0.502871	3.139088	-0.199626
2.	6.	0.	-0.500320	2.881238	1.185469
3.	6.	0.	-0.257775	1.590667	1.658390
4.	6.	0.	-0.015783	0.578133	0.737312
5.	6.	0.	0.014527	0.805039	-0.648726
6.	6.	0.	-0.243605	2.114176	-1.104682
7.	8.	0.	0.196155	-0.671122	1.251053
8.	6.	0.	0.415865	-1.738922	0.421612
9.	6.	0.	0.452173	-1.632065	-0.933140
10.	6.	0.	0.298378	-0.310363	-1.562581
11.	6.	0.	0.559223	-2.995736	1.228119
12.	6.	0.	-0.806398	-3.607009	1.631602
13.	8.	0.	-1.555040	-3.998251	0.484157
14.	6.	0.	0.700913	-2.808631	-1.845899
15.	8.	0.	0.406882	-0.141893	-2.781705
16.	6.	0.	-0.395951	2.462109	-2.575758
17.	8.	0.	0.692072	2.840574	-3.276925
18.	8.	0.	-1.495156	2.556150	-3.066024
19.	8.	0.	-0.733277	3.820758	2.134913
20.	6.	0.	-1.026988	5.153582	1.726117
21.	6.	0.	2.010227	2.579507	-2.780203
22.	6.	0.	-0.627284	-4.855118	2.484981
23.	1.	0.	-0.727658	4.128875	-0.576325
24.	1.	0.	-0.269302	1.383050	2.721325

25.	1.	0.	1.121656	-3.741223	0.661236
26.	1.	0.	1.125645	-2.759168	2.135652
27.	1.	0.	-1.362363	-2.850174	2.207454
28.	1.	0.	-1.761364	-3.203698	-0.027366
29.	1.	0.	1.771914	-3.030729	-1.928784
30.	1.	0.	0.348983	-2.554690	-2.847338
31.	1.	0.	0.189423	-3.708615	-1.500153
32.	1.	0.	-1.183638	5.716547	2.646459
33.	1.	0.	-1.936394	5.194931	1.115700
34.	1.	0.	-0.193127	5.595940	1.168456
35.	1.	0.	2.222205	1.509573	-2.830581
36.	1.	0.	2.137939	2.942829	-1.755813
37.	1.	0.	2.684807	3.123961	-3.442015
38.	1.	0.	-0.068356	-5.616267	1.931208
39.	1.	0.	-1.603158	-5.272005	2.746031
40.	1.	0.	-0.086179	-4.624048	3.407399

Conformer 1-4

1-4		Standard Orientation (Ångstroms)			
Center number	Atom number	Type	X	Y	Z
1.	6.	0.	-0.522445	3.077948	-0.351415
2.	6.	0.	-0.580006	2.825909	1.033457
3.	6.	0.	-0.405917	1.528128	1.518513
4.	6.	0.	-0.182284	0.502713	0.607688
5.	6.	0.	-0.145724	0.713918	-0.779178
6.	6.	0.	-0.313784	2.033412	-1.246956
7.	8.	0.	0.003303	-0.748946	1.132511
8.	6.	0.	0.245861	-1.835704	0.319839
9.	6.	0.	0.307639	-1.728710	-1.032503
10.	6.	0.	0.074011	-0.426252	-1.677347
11.	6.	0.	0.431167	-3.062240	1.164726
12.	6.	0.	1.709240	-3.036910	2.045161
13.	8.	0.	1.589042	-2.116801	3.121206
14.	6.	0.	0.574993	-2.871732	-1.974879
15.	8.	0.	0.050832	-0.305046	-2.906584
16.	6.	0.	-0.144989	2.422305	-2.706018
17.	8.	0.	-1.217184	2.387990	-3.523045
18.	8.	0.	0.891939	2.908580	-3.087493
19.	8.	0.	-0.792225	3.779673	1.972355
20.	6.	0.	-0.949480	5.133158	1.555154
21.	6.	0.	-2.399329	1.668550	-3.152542

22.	6.	0.	1.975981	-4.402165	2.667813
23.	1.	0.	-0.619555	4.086369	-0.733155
24.	1.	0.	-0.441176	1.332527	2.583521
25.	1.	0.	-0.429923	-3.164473	1.836820
26.	1.	0.	0.453398	-3.942532	0.521239
27.	1.	0.	2.561860	-2.764597	1.400203
28.	1.	0.	1.303496	-1.269023	2.755045
29.	1.	0.	1.395294	-2.607250	-2.648635
30.	1.	0.	-0.296780	-3.048933	-2.613464
31.	1.	0.	0.829023	-3.800778	-1.464815
32.	1.	0.	-1.101053	5.707460	2.469237
33.	1.	0.	-1.822285	5.253027	0.902706
34.	1.	0.	-0.054924	5.500407	1.039388
35.	1.	0.	-2.749811	1.948291	-2.154284
36.	1.	0.	-2.210887	0.594060	-3.199052
37.	1.	0.	-3.153543	1.946384	-3.889707
38.	1.	0.	1.116849	-4.723124	3.265537
39.	1.	0.	2.845015	-4.341172	3.327828
40.	1.	0.	2.173361	-5.155419	1.899124

Conformer **1-5**

**1-5**		Standard Orientation (Ångstroms)			
Center number	Atom number	Type	X	Y	Z
1.	6.	0.	-0.475427	3.319007	-0.348568
2.	6.	0.	-0.458718	3.023290	1.031720
3.	6.	0.	-0.238983	1.719860	1.468456
4.	6.	0.	-0.024892	0.729335	0.503942
5.	6.	0.	-0.004831	0.994870	-0.869130
6.	6.	0.	-0.250473	2.326602	-1.286339
7.	8.	0.	0.174103	-0.533567	0.981941
8.	6.	0.	0.366452	-1.581411	0.118194
9.	6.	0.	0.407427	-1.435418	-1.229303
10.	6.	0.	0.265629	-0.094586	-1.817845
11.	6.	0.	0.513654	-2.858529	0.892453
12.	6.	0.	-0.824154	-3.411567	1.413145
13.	8.	0.	-1.584325	-3.757115	0.256251
14.	6.	0.	0.604389	-2.597940	-2.170051
15.	8.	0.	0.376875	0.116252	-3.030777
16.	6.	0.	-0.423711	2.711062	-2.746010
17.	8.	0.	0.653946	3.116986	-3.449023

18.	8.	0.	-1.528199	2.803905	-3.223601
19.	8.	0.	-0.679041	4.081089	1.851569
20.	6.	0.	-0.705426	3.856998	3.255795
21.	6.	0.	1.977730	2.844264	-2.974845
22.	6.	0.	-0.620937	-4.619711	2.327477
23.	1.	0.	-0.688621	4.338371	-0.650966
24.	1.	0.	-0.232669	1.440791	2.513564
25.	1.	0.	0.983499	-3.620913	0.266682
26.	1.	0.	1.171930	-2.670627	1.748108
27.	1.	0.	-1.330933	-2.611520	1.975698
28.	1.	0.	-2.471286	-4.012760	0.540594
29.	1.	0.	1.622749	-3.001034	-2.113250
30.	1.	0.	0.444504	-2.247789	-3.190927
31.	1.	0.	-0.097141	-3.405981	-1.950529
32.	1.	0.	-0.898933	4.829110	3.709828
33.	1.	0.	0.255138	3.471248	3.618650
34.	1.	0.	-1.504766	3.159886	3.534981
35.	1.	0.	2.183124	1.773610	-3.038379
36.	1.	0.	2.123403	3.197637	-1.949363
37.	1.	0.	2.645202	3.391397	-3.641756
38.	1.	0.	-0.095173	-5.418117	1.794258
39.	1.	0.	-1.585932	-5.015234	2.663749
40.	1.	0.	-0.043185	-4.352753	3.218607

Table S3. Gibbs free energies[a] and equilibrium populations[b] of low-energy conformers of phomochromenone G (**4**)

Conformers	In gas	
	G[a]	*P* (%)[b]
**4-1**	-911928.7694478	18.09
**4-2**	-911929.27459335	42.48
**4-3**	-911928.57052713	12.93
**4-4**	-911928.24986952	7.52
**4-5**	-911928.79768575	18.98

[a]B3LYP/6-31G(d,p), in kcal/mol. [b]From *G* values at 298.15K.

Table S4. Cartesian coordinates for the low-energy reoptimized MMFF conformers of phomochromenone G (**4**) at B3LYP/6-31G(d,p) level of theory in gas

Conformer **4-1**

**4-1**		Standard Orientation (Ångstroms)			
Center number	Atomic number	Atomic Type	X	Y	Z
1.	6.	0.	3.646266	0.825284	0.032879

2.	6.	0.	4.102507	-0.490096	0.156935
3.	6.	0.	3.204589	-1.565565	0.116914
4.	6.	0.	1.847310	-1.288709	-0.078917
5.	6.	0.	1.364725	0.024932	-0.217533
6.	6.	0.	2.288278	1.087585	-0.129451
7.	8.	0.	1.022888	-2.379789	-0.135038
8.	6.	0.	-0.306927	-2.199398	-0.316335
9.	6.	0.	-0.878790	-0.987074	-0.506176
10.	6.	0.	-0.059786	0.220779	-0.555959
11.	8.	0.	-0.499741	1.325498	-0.900178
12.	6.	0.	1.892068	2.537034	-0.182675
13.	8.	0.	1.067986	2.863390	0.828258
14.	8.	0.	2.357462	3.331087	-0.972154
15.	8.	0.	5.402126	-0.875042	0.333141
16.	6.	0.	6.411517	0.131088	0.367307
17.	6.	0.	0.574772	4.213680	0.799153
18.	6.	0.	-1.070693	-3.484702	-0.338782
19.	6.	0.	-2.565816	-3.223301	-0.092286
20.	8.	0.	-2.996661	-2.141876	-0.932729
21.	6.	0.	-2.371085	-0.884634	-0.747900
22.	8.	0.	3.627012	-2.846899	0.244430
23.	8.	0.	-2.916657	-0.159961	0.334600
24.	6.	0.	-2.907642	-3.034832	1.388074
25.	6.	0.	-4.179618	0.492249	0.067382
26.	6.	0.	-3.936985	1.975957	-0.278851
27.	6.	0.	-5.083254	0.274638	1.275863
28.	8.	0.	-3.183081	2.084490	-1.477223
29.	6.	0.	-3.295173	2.780465	0.855374
30.	1.	0.	4.337671	1.657745	0.068747
31.	1.	0.	7.356099	-0.397022	0.498275
32.	1.	0.	6.257307	0.817182	1.207823
33.	1.	0.	6.435909	0.698751	-0.569509
34.	1.	0.	-0.028915	4.324277	1.699338
35.	1.	0.	1.401259	4.928004	0.796264
36.	1.	0.	-0.038494	4.366068	-0.091993
37.	1.	0.	-0.926064	-3.956858	-1.319173
38.	1.	0.	-0.656378	-4.168313	0.409436
39.	1.	0.	-3.131122	-4.081054	-0.469856
40.	1.	0.	-2.561657	-0.347542	-1.682561
41.	1.	0.	4.590773	-2.814076	0.353467
42.	1.	0.	-3.973654	-2.824393	1.500079
43.	1.	0.	-2.354341	-2.206911	1.835306
44.	1.	0.	-2.678713	-3.953733	1.939259

45.	1.	0.	-4.634834	0.024161	-0.814080
46.	1.	0.	-4.921523	2.407173	-0.509259
47.	1.	0.	-4.586446	0.592087	2.196849
48.	1.	0.	-5.341430	-0.782712	1.376759
49.	1.	0.	-6.012756	0.844451	1.171017
50.	1.	0.	-2.253482	1.869924	-1.266682
51.	1.	0.	-2.334919	2.337986	1.136402
52.	1.	0.	-3.120543	3.804799	0.514218
53.	1.	0.	-3.931992	2.820544	1.744928

Conformer **4-2**

**4-2**		Standard Orientation (Ångstroms)			
Center number	Atom number	Type	X	Y	Z
1.	6.	0.	-3.691043	-0.732659	-0.042978
2.	6.	0.	-4.106963	0.584792	0.167230
3.	6.	0.	-3.171968	1.625997	0.243738
4.	6.	0.	-1.817963	1.314586	0.078431
5.	6.	0.	-1.374712	-0.001252	-0.143826
6.	6.	0.	-2.337150	-1.032317	-0.175120
7.	8.	0.	-0.957936	2.378731	0.118891
8.	6.	0.	0.374058	2.161393	0.004277
9.	6.	0.	0.915524	0.939474	-0.218418
10.	6.	0.	0.055389	-0.218873	-0.449693
11.	8.	0.	0.466054	-1.291136	-0.909993
12.	6.	0.	-1.994096	-2.487457	-0.328496
13.	8.	0.	-1.182271	-2.916001	0.653220
14.	8.	0.	-2.489390	-3.206352	-1.170751
15.	8.	0.	-5.399006	1.003163	0.323493
16.	6.	0.	-6.442329	0.035775	0.235620
17.	6.	0.	-0.748372	-4.281301	0.530824
18.	6.	0.	1.179104	3.420817	0.079943
19.	6.	0.	2.549866	3.180931	-0.571314
20.	8.	0.	3.111915	1.997643	-0.012825
21.	6.	0.	2.421578	0.790771	-0.339204
22.	8.	0.	-3.555356	2.909216	0.450568
23.	8.	0.	2.835097	-0.181513	0.563573
24.	6.	0.	3.535226	4.312768	-0.327569
25.	6.	0.	4.113079	-0.797471	0.280311
26.	6.	0.	3.862941	-2.215727	-0.268540

27.	6.	0.	4.956195	-0.727319	1.547366
28.	8.	0.	3.149174	-2.135183	-1.493145
29.	6.	0.	3.163449	-3.149301	0.723480
30.	1.	0.	-4.410441	-1.539812	-0.100355
31.	1.	0.	-7.373405	0.586797	0.369110
32.	1.	0.	-6.348880	-0.719375	1.024257
33.	1.	0.	-6.444316	-0.455538	-0.743701
34.	1.	0.	-0.140107	-4.475053	1.413616
35.	1.	0.	-1.605425	-4.957762	0.493995
36.	1.	0.	-0.152498	-4.402926	-0.376617
37.	1.	0.	0.629152	4.227150	-0.417949
38.	1.	0.	1.314290	3.715123	1.128855
39.	1.	0.	2.403056	3.048298	-1.656655
40.	1.	0.	2.683805	0.497137	-1.368750
41.	1.	0.	-4.523246	2.903721	0.521085
42.	1.	0.	4.488800	4.093223	-0.814111
43.	1.	0.	3.716839	4.432532	0.744866
44.	1.	0.	3.148608	5.256161	-0.725533
45.	1.	0.	4.607366	-0.228003	-0.516081
46.	1.	0.	4.845552	-2.636507	-0.524391
47.	1.	0.	4.435821	-1.189989	2.390445
48.	1.	0.	5.154854	0.316538	1.803421
49.	1.	0.	5.913130	-1.241487	1.406683
50.	1.	0.	2.229875	-1.879686	-1.283343
51.	1.	0.	2.980875	-4.113774	0.241285
52.	1.	0.	3.766603	-3.322874	1.620483
53.	1.	0.	2.202576	-2.724195	1.028767

Conformer **4-3**

**4-3**		Standard Orientation (Ångstroms)			
Center number	Atom number	Type	X	Y	Z
1.	6.	0.	3.665979	0.687349	0.318113
2.	6.	0.	4.051014	-0.656487	0.360135
3.	6.	0.	3.117046	-1.677122	0.134063
4.	6.	0.	1.787737	-1.315636	-0.107304
5.	6.	0.	1.372948	0.026988	-0.139683
6.	6.	0.	2.344736	1.031616	0.047043
7.	8.	0.	0.916127	-2.351317	-0.308126
8.	6.	0.	-0.393592	-2.086507	-0.526368

9.	6.	0.	-0.920111	-0.838508	-0.510943
10.	6.	0.	-0.066337	0.323365	-0.282906
11.	8.	0.	-0.495645	1.482918	-0.212021
12.	6.	0.	2.026147	2.500157	-0.040651
13.	8.	0.	1.690839	2.850107	-1.294599
14.	8.	0.	2.166007	3.276750	0.879171
15.	8.	0.	5.313308	-1.121507	0.602011
16.	6.	0.	6.347535	-0.177930	0.869834
17.	6.	0.	1.239575	4.206337	-1.452301
18.	6.	0.	-1.187092	-3.317977	-0.823998
19.	6.	0.	-2.688306	-3.061624	-0.611618
20.	8.	0.	-3.045832	-1.815165	-1.228765
21.	6.	0.	-2.405066	-0.644516	-0.751450
22.	8.	0.	3.474301	-2.984512	0.159555
23.	8.	0.	-2.970390	-0.167228	0.450668
24.	6.	0.	-3.113784	-3.179311	0.854343
25.	6.	0.	-4.210141	0.564529	0.301195
26.	6.	0.	-3.926464	2.081131	0.323134
27.	6.	0.	-5.167683	0.096494	1.391453
28.	8.	0.	-3.097679	2.437985	-0.774190
29.	6.	0.	-3.343800	2.585896	1.645847
30.	1.	0.	4.389324	1.475397	0.486635
31.	1.	0.	7.249053	-0.764487	1.047486
32.	1.	0.	6.117548	0.418724	1.759542
33.	1.	0.	6.507823	0.486945	0.013559
34.	1.	0.	1.099057	4.346152	-2.523736
35.	1.	0.	0.292519	4.341154	-0.924249
36.	1.	0.	1.978407	4.909819	-1.062407
37.	1.	0.	-0.995854	-3.601467	-1.867297
38.	1.	0.	-0.830182	-4.142891	-0.198949
39.	1.	0.	-3.248008	-3.802088	-1.191622
40.	1.	0.	-2.560188	0.091253	-1.547338
41.	1.	0.	4.424712	-3.011166	0.353651
42.	1.	0.	-4.179221	-2.959583	0.951899
43.	1.	0.	-2.568907	-2.486884	1.498632
44.	1.	0.	-2.940112	-4.202782	1.205038
45.	1.	0.	-4.637065	0.328008	-0.681207
46.	1.	0.	-4.889298	2.580131	0.142641
47.	1.	0.	-4.700224	0.166922	2.377448
48.	1.	0.	-5.459723	-0.943895	1.227170
49.	1.	0.	-6.075464	0.709277	1.396024
50.	1.	0.	-2.190145	2.148002	-0.557025
51.	1.	0.	-2.413316	2.059579	1.877212

52.	1.	0.	-3.123205	3.653277	1.558758
53.	1.	0.	-4.037853	2.446883	2.480876

Conformer **4-4**

**4-4**		Standard Orientation (Ångstroms)			
Center number	Atom number	Type	X	Y	Z
1.	6.	0.	3.630391	1.038074	0.012030
2.	6.	0.	4.179025	-0.244800	0.105838
3.	6.	0.	3.360806	-1.382641	0.053769
4.	6.	0.	1.984944	-1.201666	-0.120679
5.	6.	0.	1.412118	0.078515	-0.229120
6.	6.	0.	2.255757	1.204157	-0.130814
7.	8.	0.	1.235328	-2.347396	-0.180519
8.	6.	0.	-0.106672	-2.254012	-0.340283
9.	6.	0.	-0.756442	-1.079054	-0.502135
10.	6.	0.	-0.028107	0.183835	-0.528649
11.	8.	0.	-0.566893	1.259453	-0.821035
12.	6.	0.	1.733655	2.615858	-0.162090
13.	8.	0.	0.923521	2.857472	0.882677
14.	8.	0.	2.093368	3.443882	-0.971236
15.	8.	0.	5.504614	-0.539580	0.262192
16.	6.	0.	6.438456	0.535454	0.326584
17.	6.	0.	0.227845	4.118606	0.863810
18.	6.	0.	-0.796816	-3.580725	-0.362919
19.	6.	0.	-2.300465	-3.406602	-0.080507
20.	8.	0.	-2.816409	-2.343958	-0.898784
21.	6.	0.	-2.254041	-1.057548	-0.713078
22.	8.	0.	3.877709	-2.631735	0.153410
23.	8.	0.	-2.800253	-0.369815	0.387068
24.	6.	0.	-2.622928	-3.248235	1.407509
25.	6.	0.	-4.091120	0.252327	0.184150
26.	6.	0.	-3.897266	1.603533	-0.546396
27.	6.	0.	-4.697572	0.393247	1.573695
28.	8.	0.	-2.988492	2.429938	0.161204
29.	6.	0.	-5.200126	2.375712	-0.730305
30.	1.	0.	4.262217	1.916299	0.058065
31.	1.	0.	7.418658	0.073658	0.446169
32.	1.	0.	6.233875	1.185602	1.184681

33.	1.	0.	6.423197	1.128442	-0.594634
34.	1.	0.	-0.137038	4.270623	1.879315
35.	1.	0.	0.898449	4.925248	0.563437
36.	1.	0.	-0.614677	4.052805	0.170474
37.	1.	0.	-0.648281	-4.035635	-1.350811
38.	1.	0.	-0.330413	-4.247717	0.369515
39.	1.	0.	-2.823590	-4.292129	-0.454564
40.	1.	0.	-2.481908	-0.528733	-1.646205
41.	1.	0.	4.837384	-2.529620	0.255190
42.	1.	0.	-3.695221	-3.083125	1.537512
43.	1.	0.	-2.097034	-2.402450	1.854374
44.	1.	0.	-2.346433	-4.162028	1.945093
45.	1.	0.	-4.712552	-0.410981	-0.434634
46.	1.	0.	-3.492159	1.382084	-1.547282
47.	1.	0.	-4.111110	1.106760	2.157942
48.	1.	0.	-4.692920	-0.572697	2.084969
49.	1.	0.	-5.729946	0.748935	1.519685
50.	1.	0.	-2.116680	2.009859	0.053748
51.	1.	0.	-5.595793	2.705628	0.234310
52.	1.	0.	-5.017727	3.265246	-1.338928
53.	1.	0.	-5.958885	1.763631	-1.229692

Conformer **4-5**

**4-5**		Standard Orientation (Ångstroms)			
Center number	Atom number	Type	X	Y	Z
1.	6.	0.	-3.671523	-0.966586	-0.071148
2.	6.	0.	-4.189948	0.321447	0.092504
3.	6.	0.	-3.340032	1.434594	0.163540
4.	6.	0.	-1.962456	1.224808	0.041138
5.	6.	0.	-1.419022	-0.059669	-0.136188
6.	6.	0.	-2.296314	-1.163685	-0.162357
7.	8.	0.	-1.181393	2.349909	0.080198
8.	6.	0.	0.165614	2.226122	0.002441
9.	6.	0.	0.796612	1.041350	-0.177465
10.	6.	0.	0.029557	-0.181894	-0.391933
11.	8.	0.	0.538045	-1.233091	-0.800501
12.	6.	0.	-1.818071	-2.585930	-0.274304
13.	8.	0.	-1.016009	-2.915767	0.751955
14.	8.	0.	-2.204094	-3.354572	-1.129284

15.	8.	0.	-5.513650	0.644152	0.206234
16.	6.	0.	-6.478952	-0.401057	0.119382
17.	6.	0.	-0.375104	-4.202355	0.660741
18.	6.	0.	0.884808	3.537095	0.073168
19.	6.	0.	2.288679	3.375249	-0.531200
20.	8.	0.	2.914037	2.241469	0.063598
21.	6.	0.	2.311245	0.989967	-0.262484
22.	8.	0.	-3.827712	2.688668	0.326495
23.	8.	0.	2.759314	0.052811	0.657950
24.	6.	0.	3.189113	4.574549	-0.280853
25.	6.	0.	4.044429	-0.559861	0.388334
26.	6.	0.	3.827376	-1.759931	-0.563316
27.	6.	0.	4.606838	-0.943927	1.749228
28.	8.	0.	2.878171	-2.660440	-0.019585
29.	6.	0.	5.107812	-2.535418	-0.856108
30.	1.	0.	-4.326858	-1.827076	-0.121448
31.	1.	0.	-7.451718	0.080857	0.218673
32.	1.	0.	-6.348068	-1.128804	0.928209
33.	1.	0.	-6.420956	-0.913518	-0.847353
34.	1.	0.	0.025016	-4.402964	1.654145
35.	1.	0.	-1.091374	-4.970451	0.364121
36.	1.	0.	0.439428	-4.150296	-0.066238
37.	1.	0.	0.301774	4.296534	-0.459649
38.	1.	0.	0.966769	3.861114	1.118839
39.	1.	0.	2.183369	3.214815	-1.617839
40.	1.	0.	2.608170	0.713532	-1.289548
41.	1.	0.	-4.793713	2.608421	0.373685
42.	1.	0.	4.169582	4.411275	-0.735014
43.	1.	0.	3.329852	4.724389	0.793901
44.	1.	0.	2.752814	5.483027	-0.707657
45.	1.	0.	4.698069	0.179826	-0.094996
46.	1.	0.	3.448111	-1.354038	-1.516052
47.	1.	0.	3.983083	-1.721568	2.196803
48.	1.	0.	4.615716	-0.071247	2.406787
49.	1.	0.	5.628736	-1.322955	1.659994
50.	1.	0.	2.026631	-2.189453	-0.058071
51.	1.	0.	4.909138	-3.303648	-1.608015
52.	1.	0.	5.896705	-1.875983	-1.233799
53.	1.	0.	5.472081	-3.036118	0.045306
